# WHO Environmental Noise Guidelines for the European Region: A Systematic Review of Transport Noise Interventions and Their Impacts on Health

**DOI:** 10.3390/ijerph14080873

**Published:** 2017-08-03

**Authors:** Alan Lex Brown, Irene van Kamp

**Affiliations:** 1 Griffith School of Environment/Cities Research Institute, Griffith University, Brisbane 4075, Australia; 2 Centre for Sustainability, Environment and Health, National Institute for Public Health and the Environment (RIVM), 3720BA Bilthoven, The Netherlands; Irene.van.kamp@rivm.nl

**Keywords:** transport noise, interventions, health effects

## Abstract

This paper describes a systematic review (1980–2014) of evidence on effects of transport noise interventions on human health. The sources are road traffic, railways, and air traffic. Health outcomes include sleep disturbance, annoyance, cognitive impairment of children and cardiovascular diseases. A conceptual framework to classify noise interventions and health effects was developed. Evidence was thinly spread across source types, outcomes, and intervention types. Further, diverse intervention study designs, methods of analyses, exposure levels, and changes in exposure do not allow a meta-analysis of the association between changes in noise level and health outcomes, and risk of bias in most studies was high. However, 43 individual transport noise intervention studies were examined (33 road traffic; 7 air traffic; 3 rail) as to whether the intervention was associated with a change in health outcome. Results showed that many of the interventions were associated with changes in health outcomes irrespective of the source type, the outcome or intervention type (source, path or infrastructure). For road traffic sources and the annoyance outcome, the expected effect-size can be estimated from an appropriate exposure–response function, though the change in annoyance in most studies was larger than could be expected based on noise level change.

## 1. Introduction

This paper systematically reviews the literature from 1980 to 2014 on evidence of the effects of transport noise interventions on human health. A wide range of noise interventions, noise management, or noise control, actions are included, and the source types considered in this review were road traffic, railways, and air traffic. The intent of both exposure-related, and non-exposure-related, interventions is to change (generally reduce) the adverse health outcomes from noise, and the health outcomes reported here include sleep disturbance, annoyance, cognitive impairment of children, and cardiovascular diseases. Exposure-related actions aim to change the level of noise exposure of people, usually as measured at the external façade of their dwellings. Non-exposure related actions such as communication or education are directed at changing health outcomes but do not include changing people’s exposure. The different noise sources, and the different types of interventions possible for each noise source, introduce considerable complexity to this review, and a structure that provides a conceptual framework for considering interventions and health effects is presented in the next section.

## 2. A Framework for Noise Interventions 

A conceptual model by which to consider noise interventions and their health effects was first reported by Brown and van Kamp [1]. The model built on related frameworks from the air pollution field that have been utilised to evaluate whether actions taken to improve air quality have resulted in reduced health effects—so-called *air pollution accountability research* [2,3,4,5]. These air pollution frameworks have an emphasis on ambient concentrations of the pollutants, but this is not appropriate for environmental noise where exposure of people is always strongly influenced by the length and nature of the propagation paths from sources to receivers, and hence highly dependent on the disposition of receivers relative to the sources. For noise interventions, the propagation path thus needs to figure as a significant component of the system between sources and the human receivers, and this has been incorporated in the basic systems model between environmental noise sources and human health. This framework is generic to all sources of environmental noise.

Another difference is that air pollution accountability research has tended to focus on regulatory interventions directed at reducing emissions; examining whether this type of intervention consequently reduces ambient concentrations over time. While regulatory intervention is also used in managing environmental noise, for example by control of aircraft or road vehicle source levels, this is only one of a range of possible environmental noise interventions [6] (Chapter 5). Environmental noise management, or environmental noise control, often involves technical interventions that include not only reduction of source emissions but also alteration of the transmission path, for example by the positioning of outdoor barriers between source and receivers, and changes in the acoustic properties of building envelopes to reduce levels at receivers. It also includes other source-related changes such as time restrictions on operations of sources, or changes in infrastructure. Examples of the latter include the opening or closure of new roadways and railway lines, bypass roadways, or the opening of new airports/runways and consequent rearrangement of air traffic load on flight paths. Environmental noise management has also utilised interventions that promote change that reduces peoples’ exposures or that is directed at mitigating their adverse reactions to exposure. Communicating an authority’s intent to make changes, e.g., with respect to flight paths, is an example of the latter.

Based on the available intervention literature, and the experience of many decades of noise management, five broad categories of transport noise intervention were identified and are listed in Table 1. Terminology for two of the technical interventions has been borrowed from the environmental noise control field (*source interventions* and *path interventions*). The third category of intervention is termed *new/closed infrastructure.* The fourth category is termed *other physical interventions*, and the fifth category referred to as *education/communication interventions*. The categories and sub-categories of these intervention types are largely self-explanatory, but they are also illustrated by the examples included in Table 1. Such categorisation of interventions is necessary as compilation of evidence regarding outcomes from interventions may only be appropriate when the evidence comes from studies that belong to the same category. This framework provides a systematic and comprehensive basis for this (and any future) work with respect to the effects of noise interventions.

**Figure 1:**
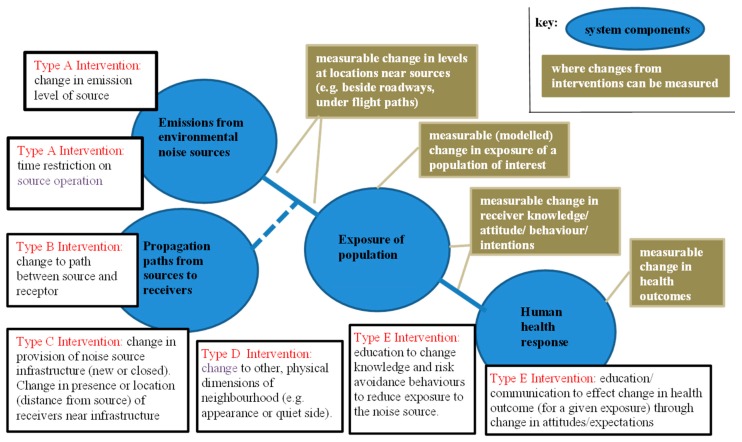
Intervention framework showing: system components of the path between environmental noise and human health, where different types of noise intervention potentially act along that path, and points along the pathway where changes resulting from interventions can be measured.

The framework for considering noise interventions and related health effects is in Figure 1. It shows where different categories of interventions fit along the system pathway between noise sources and human outcomes. It also shows different measurement points along the pathway where changes relevant to human outcomes can be measured. This framework provides a systematic and comprehensive basis for this, and any future work with respect to the effects of interventions in environmental noise of all source types. Note that not all of the interventions types included in the framework are represented in the individual studies identified in the literature search described below.

## 3. Measurement of the Health Outcomes of an Intervention

Noise interventions are presumed to result in changes in various outcomes along the system pathway between noise sources and human health responses.

Evidence of the effect of any noise intervention on human health can best be examined in studies in which the effect of the intervention has been reported directly in terms of a change in health outcomes. The availability of a measured change in health outcomes in an intervention study was the primary basis for inclusion of a study in this review.

However, on the assumption that there is a well-established link between exposure and particular health outcomes, it is not necessary to evaluate interventions only by means of change in measured health outcome. Evaluation by the *intermediate outcome* of change in exposure of a population of interest is also appropriate as change in exposure can be presumed to result in changes in health outcome. Thus, individual studies that reported a change in the exposures of the population of interest were also included.

In addition, certain interventions for environmental noise, directed at changing knowledge or perceptions, may result in change in exposure of a group. They also may result directly in changes in health outcomes—as where a group may report lower annoyance scores from a transport source if authorities have undertaken a program of communication and explanation regarding the noise. Thus, studies where the intervention was designed to educate or change behaviour or perceptions were also considered.

Figure 1 shows, on the intervention framework, where each of the change in *health outcomes*, the intermediate change in *exposure outcomes*, and the *change in knowledge/attitude outcomes* could potentially be measured.

We note, however, that there are many examples in the literature of noise management or noise control where the effect of a noise intervention is reported solely as change in the level of noise at or near the source. For example, the effects of motor vehicle source limit regulations, or of limits on aircraft noise emission resulting from certification requirements, may be reported as changes in noise levels emitted by these sources. Equally, the effect of a path intervention through construction of a noise barrier near a roadway may be reported as the change in level immediately behind the barrier—not as a change in exposure levels for some affected population. Similarly, after an intervention involving modification to airport flight paths, the effect may be reported as changes in noise levels at particular points on the ground—again not as a change in exposure levels of an affected population. These types of outcomes are indicated on Figure 1 as *measurable change in levels at locations near sources*. Studies of this sort that report only change in levels near sources, rather than changes in people’s exposure, cannot be utilised to elucidate the relationship between interventions and their health consequences.

## 4. Methods

### 4.1. Search Strategy for the Identification of Studies

Five prior narrative reviews on environmental noise interventions were located. These are listed in Supplementary File 1.

To identify individual studies, we performed search runs on the following data sets:
SBASScopusME66MEDLINENLMEM74EMBASE2014 Elsevier B.V.PI67PsycINFOAM. PSYCH. ASSN. 2010IN73Social SciSearchThomson ReutersIS74SciSearchThomson ReutersBA70BIOSIS PreviewsThomson Reuters

The search string was refined and adapted for the different data bases and is available (Supplementary File 2). The search was restricted to publication years 1980–2014.

### 4.2. Inclusion Criteria

Papers were read independently by the two authors, applying the following inclusion criteria. A study was retained for further analysis if the following conditions were met:

1. It dealt with noise sources as defined in the Study Protocol … *rail*, *road*, *aircraft*
2. It reported the nature of an intervention of any Type A through E
3. It specified (for intervention Types A–C), the change in exposure, usually as levels before and after the intervention
4. The intervention (for intervention Types A–C) was not temporary or laboratory-based
5. It reported:
  - before and after health outcomes OR
  - before and after exposures of a target population OR
  - for interventions Type E, before and after knowledge/attitudes/behaviour OR
  - a comparison of two exposure conditions with variation in some other relevant factor (e.g., quiet side).

A list of the papers that were excluded, with brief explanation of the reasons for exclusion, is available (Supplementary File 3).

### 4.3. Data Extraction and Synthesis of Findings Across Studies

It will be shown below that, overall, there is a relatively small number of studies available on the health effect of interventions. Further, when individual studies are grouped according to noise source categories, health outcome categories, and intervention categories, the number of studies within most of the categories is very small. Further, even in categories with more than two or three studies, these studies tend to be very different from each other.

Differences between studies include the magnitude of the change in exposure that results from the intervention; the distribution of the magnitude of change in exposure across the study sample (the nature of many of the interventions is such that the change in noise level exposure varies across a study site. For example, people close to the roadway in a barrier intervention experience a large reduction in noise levels, but, further from the barrier, they experience less change, out to eventually a zero change. Several of the studies like this reported only wide bands of noise level magnitude change; others reported the mean magnitude change for (sub) groups of respondents. This allows for relating the observed outcomes only to some averaged change in exposure rather than to actual individual changes in exposure); the exposure levels before the intervention; the study design; and the approach to data analysis. Differences in the latter include reporting response scores for groups/subgroups as measures of central tendency (mean or median)—which cannot then be converted to, say, percentages highly annoyed as is commonly used in other studies. Some studies analyzed and reported outcome responses as logistic regressions of individual responses on exposures, with no reporting of effects at group or subgroup levels. Studies also utilised various noise exposure scales (for example, the traffic noise intervention studies variously reported levels on scales: L_den_, L_dn_, L_Aeq,24 h_, L_Day_, L_10,18 h_, L_10,12 h_, L_10,3 h_) and outcome response scales (sleep outcomes were reported on several different scales and in one case by wrist actigraphy. Annoyance was variously measured using annoyance, dissatisfaction, or bother, with the number of points on the response scale ranging from 4 to 10. Some studies reported Percentage Annoyed rather than Percentage Highly Annoyed). As Köhler et al. [9] note, specifically with respect to dwelling insulation but relevant to all interventions, ‘*Although the studies can be evaluated as being of good methodological quality, comparison between them is hard to make, due to difference in research groups, the way confounders are dealt with, sample size, study design…and the fact that information concerning particular characteristics is not always given…*’. Risk of bias in most individual studies was judged as high (see Supplementary File 5).

Given these differences between studies and the small number of studies within groups, it was not possible to perform a meta-analysis by means of statistical pooling the data to report the strength of association between interventions and the changes in health outcome. While we could transform some results to common scales of exposure and to common scales (and sometimes cut-offs) of response, the other differences between individual studies within each group would remain. Overall, the consequence is that we have had to seek other ways to summarise the available evidence.

We sought instead to use the evidence presented within each of the individual studies to qualitatively answer two questions with respect to the effect of environmental noise interventions. These questions were as follows:

1. Did the study demonstrate that the intervention led to a change in health outcome?
2. For source, path and infrastructure change interventions, if there was a change in health outcome, was the observed change in outcome of a magnitude at least equivalent to that which would be predicted from a relevant exposure–response function (ERF), based on the observed change in exposure?

In examining the first question, we do not assess the magnitude of the change for each individual study (but report it if available), but look instead to any evidence that health outcomes did change in association with the intervention. We include a column in the tables below to record this observation. While this question is a minimal test of the consequence of an intervention, it contributes to an important policy issue: do environmental noise interventions change health outcomes?

The second question refers to a relevant ERF. Effectively, the author(s) of each individual study specified the ERF they believed was relevant to the context of their study: either an ERF derived from before-study responses from the study area, or one that had been reported from elsewhere but was considered appropriate. Given that synthesised ERFs are, by definition, the amalgamation of a wide range of study-specific ERFs, we suggest that the approach we adopt is no less appropriate than comparing each individual study results to some normative, synthesised ERF. Further, where the comparison is with an ERF derived from the before-data of the same study, it has the advantage of controlling for many confounders in that study area. The relevant ERF used in each individual study is reported in the summary tables below. In the individual studies, the relevant ERFs (all for the annoyance outcome, except for sleep disturbance in one study) were as follows:

1. an ERF based on the responses to the before (steady-state) exposure conditions in that particular study (using grouped response data or individual responses), or sometime separate ERFs for both before and after states (5 studies used an ERF of this nature).
2. an ERF reported from similar situations to those in the particular individual study, as determined by the study authors (4 studies used an ERF of this nature).
3. a previous synthesis of ERFs. The particular ERF chosen depended on the date of the study,: namely, Schultz’s 1978 synthesis [10] (2 studies); the FICON 1992 synthesis [11] (1 study); Miedema & Vos’ 1998 synthesis [12] (2 studies); Miedema & Oudshoorn’s 2001 [13] or European Commission’s 2002 synthesis [14] (3 studies).

We compared the magnitude of the observed change in health outcome to the magnitude of the change that would be ‘predicted’ from the same change in exposure on the relevant ERF. If the observed health outcome changed similarly to the ERF-predicted change, the conclusion was that the ERF could have estimated the magnitude of the response to the intervention given the magnitude of the change in exposure. If the observed change was greater, then the study has reported an excess response to the change [15]. We include a column in the tables below recording this observation for each study. Where the magnitude of the observed change is the same as the ERF-estimated change, the slope of the observed change is parallel to the ERF; where it is greater, it is steeper than the ERF. It will be seen that there are no studies in which the slope of the observed change was found to be shallower than the ERF (which would represent an under-response to the intervention). The observations provide guidance on another important policy issue: can the magnitude of the effect of an intervention be estimated from a relevant ERF?

### 4.4. Organisation of the Review

Different source types are each considered separately in Section 6, Section 7 and Section 8 below. The review initially included hospital noise and noise from personal listening devices/music venues and other recreational sources (See Supplementary File 6), but the present paper reviews transport noise sources only. Within each section, there is an overview of the evidence available for that source type. This is followed by subsections on the different outcomes and, for each type of intervention, a narrative summary of evidence for that outcome for that intervention type, and a table listing and summarising each individual study included in that group.

## 5. Overall Search Results 

**Figure 2:**
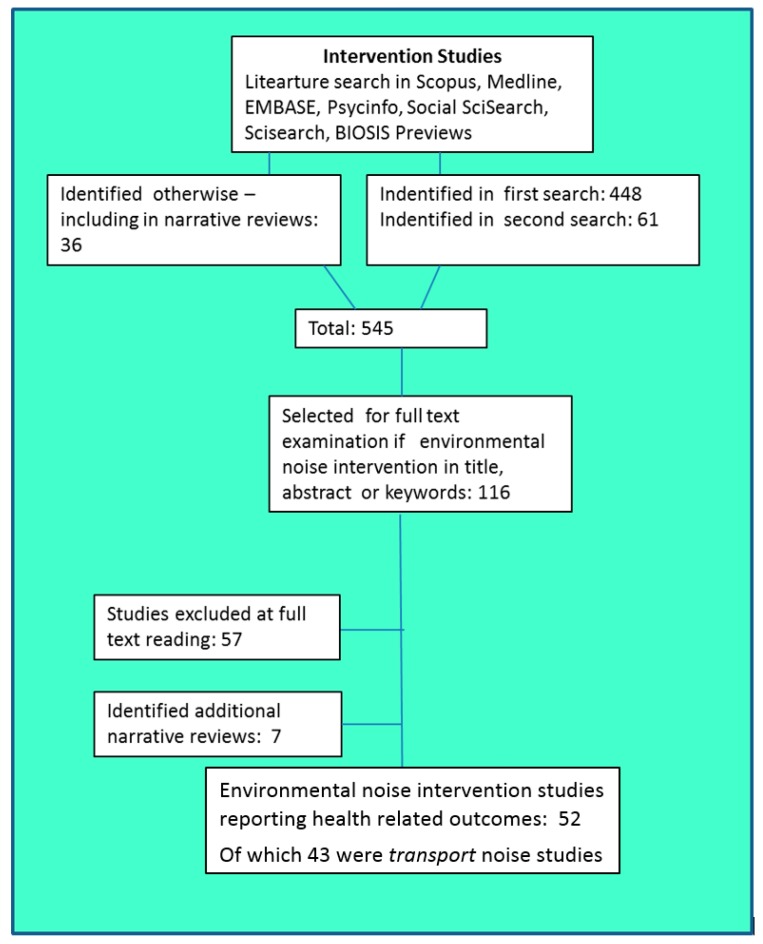
Flow diagram outlining the study selection process.

Figure 2 illustrates the literature search process. We identified over 500 studies that met our inclusion criteria. Excluding duplicates, this search resulted in 448 articles. A further 36 articles were identified through personal communications with experts and from the additional narrative reviews that had been found. After consideration of all these, we asked our professional librarian for an additional search, resulting in 61 additional articles being identified (including some duplicates).

The resulting 545 titles, keywords and abstracts were examined by each of the authors independently to identify papers that were to be read in full, based on the inclusion criteria described in Section 4.2. The result was agreement to examine the full text of 116 papers. Fifty-seven of these were excluded at full text reading, and 7 were found to be narrative reviews rather than individual studies. Of the remaining 52 noise intervention studies, 43 were transport sources and are reported on in this paper (see Supplementary File 6 for the studies of non-transport sources).

From this selection process we arrived at a small, but relevant, set of studies for each transport related noise source that linked transport noise interventions to health outcomes. The distribution of the papers, grouped across sources, outcomes, and type of intervention, is shown in Table 2. They are referred to as ‘entries’ because individual studies that reported more than one outcome are duplicated in relevant sections for each outcome. The majority of entries are for road traffic noise; fewer for aircraft noise and rail traffic noise. Previous reviews of the effects of change in noise exposure [16] have similarly reported the limited number of such studies available, and the difficulty in synthesizing information across them.

## 6. Results for Road Traffic Noise

Some 37 papers (35 after removal of duplicate reporting of the same study) describing road traffic noise interventions met the inclusion criteria—with papers counted twice if they reported results on two different outcomes. This is considerably more than the number of intervention papers reporting on each of air and rail traffic interventions (seven and three papers respectively).

For road traffic noise:

- 25 papers examined the effects of an intervention on the annoyance of adults in their dwellings
- 6 examined the effects of an intervention on sleep on adults in dwellings (several reported the effect of the intervention on both annoyance and sleep disturbance)
- 4 examined cardiovascular effects
- 2 modelled the extent of exposures to different levels of road traffic noise or the prevalence of annoyance arising from hypothetical interventions (studies modelling the effect of hypothetical interventions are considered separately from this review, in Supplementary File 4).

Under half (10) of the road traffic noise studies were source interventions; a smaller number path interventions; new or closed infrastructure; or other physical interventions.

The publication dates of the studies were approximately equally divided over three periods: 2010 to present; 2000–2009; the two decades 1980–1999. This indicates an increasing frequency of reporting of road traffic noise intervention studies, though the total number of such studies is small.

The tables below group together studies of the same health outcome from the same intervention type, summarising each of the individual studies in that group. The tables report the nature of the intervention; the study design, size and method; and the exposure levels before and after the intervention, and what is reported about the distribution of the magnitude of the changes in exposure across the study sample. The tables also show how the outcome measures of annoyance or sleep disturbance/quality changed as a consequence of the intervention, and whether the magnitude of that change was statistically significant. There is also an observation on the relationship of the observed change in response to that estimated from the same change on a relevant ERF.

### 6.1. Annoyance

#### 6.1.1. Evidence from Source Interventions

Of the source intervention studies:

- Most were where traffic flow rates on the roadway changed (including several multi-site studies). Most were a decrease in traffic flow as a consequence of provision of relief roads, but at several sites there was an increase in traffic flow. Less than half of the studies were single-site studies; the others included results from multiple roadway sites.
- 1 was where there was improved roadway resurfacing.
- 1 was a truck restriction strategy.
- 1 was a complex set of control measures including barriers, road surfaces and other measures.

All studies as presented in Table 3 were before and after designs, including two with three and four ‘after’ rounds of survey. Three of the studies included control groups. The number of participants varied between 20 and 2870.

Most of the changes experienced were reductions in noise levels, but in one study, and at a small number of sites in three multi-site studies, the change experienced were increases in level. The changes in level ranged from approximately −15 dB to +15.5 dB (various noise scales) but not uniformly across this range, with the majority of changes in the range from −5 to +5 dB. The source interventions were generally where before-conditions were high road traffic noise exposure (e.g., such as greater than L_eq,24_ of 70 dB), but several were where levels were considerably lower (by 10 to 15 dB) than this. In one study, there was very limited change in exposure resulting from reduced traffic flow and no observed change in annoyance outcomes. This study is not considered further below. In another, where there was a restriction in night-time truck traffic flow, there was zero change in energy-based noise indicators but a significant change in annoyance—postulated by the author as a response to the change in the number of noise events in the night-time traffic stream.

Apart from the two ‘no-change’ studies, the studies all found that the source intervention resulted in change in annoyance outcomes: four reported that the observed changes were statistically significant; three observations were based on data, tables or plots in the original papers, but without statistical tests.

With respect to the strength of association between the change in exposure and the change in annoyance responses, all intervention studies demonstrated that the response was of a magnitude, *at least*, as estimated by a steady-state ERF for annoyance. Two provide statistical tests that the change in response was an *excess-response* to the change—both for decreased and for increased exposures. Observations on the other studies of *excess response* were based on data, tables, or plots in the original papers, but without statistical tests.

There is only a little evidence available with respect to long-term effects of the interventions. The studies generally undertook the after-outcome measures 2 to 12 months after the intervention, but two of them also repeated the after-measure, one 12 months after the first, the other 9 years after. The limited findings from these longitudinal studies are that this excess response undergoes some attenuation, but is largely maintained out to several years.

There is only one study in this group [17] in which the exposure–response function, or the test of a change effect, was adjusted for confounding (noise sensitivity, neighbourhood quality, and association with the trucking industry). In most of the other studies the influence of confounders and potential moderators was analysed in a univariate manner or presented in exposure–response curves per subgroup. A list of the confounders variously measured in the included studies were age (2 studies); gender (2); noise sensitivity (3); length of residency (2); deprivation; general opinion of the area; attitudes towards roads, traffic, and the authorities; wish to move because of fear of accidents; type of dwelling; number in dwelling; children in house; windows open; hearing problems; awareness actions were being taken (each, 1 study).

#### 6.1.2. Evidence from Path Interventions 

Of the seven path intervention studies that are in Table 4:

- 1 was of dwelling insulation (with a repeated survey two years after the first survey reported separately);
- 3 were barrier construction (one of which was a multi-site study involving 12 sites);
- 1 was a combination of barriers and dwelling insulation;
- 1 was a full-scale building intervention, filling in gaps between existing buildings to create a barrier for dwellings further from the roadway.

All studies were before and after designs, with only the dwelling insulation study having more than one after-survey round. Most before-studies were conducted 6 months to 1–2 years before the intervention. Apart from the dwelling insulation study where the after-study was conducted some six months after insulation was installed (12 months after the before-survey) the time gap between before and after studies was much larger than for the source interventions, presumably related to the required construction time of the barrier or building refurbishments. Apart from one barrier study where the gap was 2 years, the before-after gap varied between 5 and 10 years. Three of the studies included control groups. The number of participants in the studies varied between 75 and 852.

The changes in level achieved by the interventions ranged from −3 to −13 dB (various noise scales). Apart from the dwelling insulation intervention where all participants experienced a uniform reduction in exposure of −7 dB, the variation in the change in levels experienced by respondents within any one study was wide, varying, for example, from −10 to −13 dB close to barriers, small to zero changes distant from the barriers.

All six studies (excluding the repeat after-survey study) found that the path intervention resulted in change in annoyance outcomes; three of the studies demonstrated that the observed difference in outcomes was statistically significant, the other three studies reported no statistical tests on the changes in response, but differences were observed in the data, tables, or plots in the original papers.

Four of these path intervention studies compared the change in response to that estimated by an ERF. All four showed that the response to the change was in the same direction and *at least* of the same magnitude as estimated by the ERF (statistical test reported in one study). Two of these also showed responses to the change were much steeper than the gradient of the ERF over the same exposure range, thus exhibiting an *excess response* (and another was unclear with respect to the presence of excess response). Only one study provided a statistical test of the presence of excess response.

There was one study in this group in which the exposure–response function, or the test for a change effect, was adjusted for confounding (gender, age, education level, marital status, access to a bedroom on the quiet side of the building, and noise sensitivity). In most of the other studies, the influence of confounders and potential moderators was analysed in a univariate manner. Confounders (moderators) included in this way were as follows: distance to the road (2 studies); visual aspects (2); window opening behaviour (1); other interventions such as playgrounds; access to shopping centre; opinion of residents towards noise source; coping; acceptance; window type; newcomers between surveys; negative experience before the intervention; SES (each 1 study).

#### 6.1.3. Evidence from New/Closed Infrastructure Interventions 

Of the two infrastructural intervention studies included in Table 5:

- both studies involved major changes (reductions) in road traffic flows;
- both studies combined the main intervention with other environmental improvements.

Both studies were of new road tunnel infrastructure that resulted in very large reductions in traffic and levels of noise for residents near the previously heavily trafficked surface roadway. They are distinguished from Type A interventions by the magnitude of the change in flows (e.g., traffic flow on nearest road to some participants dropped from 60,000 vehicles/d to zero).

Both studies were before and after designs using repeated measures of annoyance outcomes. Both conducted before- and after-studies approximately one year before and after the tunnel opened. Both included a control. The number of participants experiencing the change was 758 and 50 in the two studies.

Noise levels (L_Aeq,24 h_) reduced an average of −12 dB in one study (the distribution of the magnitude of the change across the sample of participants was not reported); between −11 dB and −17 dB for just under half of the respondents with the other half experiencing −3 to −5 dB reductions. In the other study, participants experienced a mean −12 dB reduction, with no information of the distribution of the magnitude of change across the sample.

Both studies demonstrated statistically significant lower annoyance responses post intervention (one tested % Annoyed; the other % Highly Annoyed and mean annoyance score) with no change in the controls.

Both studies also compared the change in outcomes to those estimated from an ERF. They reported that the after-scores in the studies matched those estimated by the ERF (though, in fact, one comparison was with an ERF of individual annoyance scores fitted only to the after exposure levels) and suggested on this basis that there was no evidence of excess response. However, both reported, but did not identify as excess response, very large changes in the before-to-after levels resulting from the interventions. This means that the response to change was not only in the same direction as estimated by the ERF, but much steeper (i.e., excess response).

None of the studies in this group adjusted for confounders in their analyses.

Confounders included otherwise were noise sensitivity (2 studies), location of bedroom (2), insulation, and window opening behaviour (each 1 study).

#### 6.1.4. Summary: Information from Other Physical Interventions 

The only studies that are available on other physical interventions (Table 6) are not intervention studies per se as they do not provide direct evidence of an intervention. Instead, they provide, by comparing responses from groups with and without the particular physical dimension of interest, indirect evidence on the magnitude of the likely effect of certain interventions (such as the provision of a quiet side to the dwelling). The existence of a ‘quiet side’ may affect exposure (depending on how a person may use different rooms in his/her house), but also may be related to perceived respite—perhaps similar to the effect resulting from the existence of green areas in the neighbourhood, or the provision of green space in the neighbourhood. Interventions of this sort could be achieved as part of comprehensive housing/roadway redesign activities over some area.

In these studies, the designs were such that participants could be similarly exposed at the most exposed façade of the dwelling but would differ on some other dimension (say the difference between the exposures on the most and least-exposed facades of the dwelling). The other physical dimensions considered in this group of studies, in addition to availability of a quiet side, included: whether bedroom or living room windows faced a quiet street (effectively a variation on the existence of a quiet side to the dwelling), the non-acoustic ‘quality’ of the space that constituted the quiet side of the dwelling (such as a courtyard); and the existence of nearby green areas. Quiet side was defined differently in the different studies: for example, 10 dB noise/quiet difference in one, L_Aeq,24 h_ less than 48 dB in another.

All studies found the presence of the particular dimension being investigated had an effect, and all but one demonstrated that this was statistically significant (for example, the difference in the percentage of at least moderately annoyed participants between homes with and without a quiet side was statistically significant). One study reported the Odds Ratio was 3.3 when adjusted for noise sensitivity (95% CI 1.35–8.01) and, when participants actually used a bedroom on the quiet side, the OR = 10.6 (CI 2.0–56). Another study showed that visual quality of the space that provided the quiet side was also relevant, with 9–13% of participants less annoyed, depending on noisy side exposure levels. The Odds Ratio for courtyard quality was 0.59 (95% CI: 0.36–0.96).

In this group, several of the studies adjusted for a large number of different confounders in their analyses (see Table 6) but others only for age, noise sensitivity, or window-closing behaviour.

### 6.2. Outcome: Sleep Disturbance

#### 6.2.1. Summary: Evidence from Source Interventions

In the one study in Table 7, there was very limited change in exposure resulting from reduced traffic flow and no observed change in sleep outcomes. This study is not considered further.

#### 6.2.2. Summary: Evidence from Path Interventions

The details of the two studies in Table 8 were reported under annoyance outcomes above (Table 4).

The studies found that the path intervention resulted in change in sleep outcomes. The percentage of people with self-reported disturbed sleep (variously measured/defined) was lower (statistically significant in one study, no tests in the other). In one of the studies, a follow-up survey two years after the intervention found that the changes observed in the initial study remained the same.

In one of the two studies in this category, the exposure response function was adjusted for confounding, including gender, age, education level, marital status, access to a bedroom on the quiet side of the building, and noise sensitivity.

#### 6.2.3. Summary: Evidence from New/Closed Infrastructure Interventions

The summary details of the two studies reported in Table 9 (new tunnels removing traffic flow on surface roadways) were also reported under annoyance outcomes above (Table 5). Subjective and objective measures of sleep quality were also assessed before and after the intervention.

Both studies demonstrated statistically significant lower reporting of various sleep disturbance indicators (or improvement in sleep compared to conditions before the intervention), post-intervention.

In one study, a remarkable finding was that the time spent in bed was significantly reduced after the intervention, suggesting increased sleep efficiency according to the authors. The group aged 48 years and over seemed to profit most from the intervention.

None of the studies adjusted for confounding in their analyses, but in one study the participants in the experimental and control groups were matched on relevant characteristics, and in this *way* the risk of confounding was minimised. Other confounders included in the study were noise sensitivity (1 study), insulation (1), quiet side (2), and window opening behaviour (2).

#### 6.2.4. Information from Other Physical Interventions

The summary details of the one study in Table 10 (new tunnels removing traffic flow on surface roadways) were reported under annoyance outcomes above (Table 6). As indicated there, this is not an intervention study per se, as it does not provide evidence of the effect of an intervention. However, it does provide indirect information on the magnitude of the likely effect of a particular intervention (such as the provision of a quiet side to the dwelling), which could be undertaken as part of a significant *housing/roadway redesign* intervention.

Subjective assessment of difficulty in falling asleep was assessed before and after the intervention. The difference in the percentage of participants reporting difficulty falling asleep ‘*at least sometimes*’ between homes with and without a quiet side was statistically significant. Absence of quiet façade results in increased reporting of this sleep parameter. The Odds Ratio for falling asleep was 5.5 (95% CI 0.7–44.1).

Confounding was adjusted for in the analyses of the ERFs including noise sensitivity, window closing behaviour, and front-façade L_den_.

### 6.3. Outcome: Cardiovascular Effects

#### Information from Other Physical Interventions

This group are, again, not intervention studies per se as they do not provide direct evidence of an intervention. However, they do provide evidence of the likely effect of a particular action (such as the provision of a quiet side to the dwelling), which could be undertaken as part of a significant housing/roadway redesign intervention. Three of the studies found changes (including in self-reported high blood pressure) with and without a quiet side—two of those were tested to be significant. One study found no change.

Confounders included age, gender, education, body mass index, physical activity at leisure, alcohol intake, family history of hypertension, and occupants per room (listed for each study in Table 11 below).

## 7. Evidence: Aircraft Noise

In the individual studies concerning aircraft noise that met the inclusion criteria, the health outcomes reported were distributed as follows:

- 4 reported effects of the intervention on the annoyance of people in their dwellings;
- 2 of these also reported effects of the intervention on sleep;
- 1 reported effects of the intervention on cognitive development in children;
- 1 modelled a hypothetical intervention in terms of the effect of the intervention on annoyance and sleep disturbance (the study modelling the effect of hypothetical interventions is considered in Supplementary File 4).

None of the studies were of source interventions; one was a path intervention involving dwelling insulation; four were new or closed infrastructure (new or abandoned or rearranged flight paths from airports).

The publication date of the aircraft studies were generally more recent that the road traffic noise interventions, with all published from 2002, with four of them in the last eight years.

The tables below group together studies of the same health outcome from the same intervention type, summarising each of the individual studies in that group. The tables report the nature of the intervention; the study design, size and method; and the exposure levels before and after the intervention, and what is reported about the distribution of the magnitude of the changes in exposure across the study sample. The tables also show how the outcome measures of annoyance or sleep outcome changed as a consequence of the intervention, and whether the magnitude of that change was statistically significant. There is also a commentary on the relationship of the observed change in response to the slope of a relevant ERF.

### 7.1. Outcome: Annoyance

#### 7.1.1. Summary: Evidence from Path Interventions

This path intervention study for aircraft noise was around five Spanish airports (Table 12). A noise insulation program (NIP) in Spain retrofitted dwellings near airports with acoustic insulation. The study was primarily interested in the overall effectiveness of this program, namely in residents’ satisfaction with the management of the process and the installation activities—but it did also assess whether there had been a change in the annoyance (and sleep disturbance) as a result of the NIP. The study demonstrated a drop in annoyance following the insulation intervention. However, no statistical tests were reported on the change in annoyance, and comparisons with other studies, and with any ERF, are not appropriate as the study used retrospective assessment by participants as the before-intervention baseline against which to compare post-intervention annoyance scores.

This study did not adjust for confounding, but reports in a descriptive manner on the influence of the aesthetics of the installed measures; and the performance of the construction company—explaining respectively 30% and 25% of the variance in satisfaction.

#### 7.1.2. Summary: Evidence from New/Closed Infrastructure Interventions 

All three studies in this group were associated with opening of new runways, closure of others, or flight path rearrangements (Table 13). Two were in Europe (Amsterdam and Zurich) and one in Canada (Vancouver). The interventions were, by and large, the introduction, or removal, of overflights, as a step change, over certain areas near the respective airports—as distinct from increases or decreases of air traffic flow along existing flight paths. Two were before and after studies, and one a panel study with four waves of survey.

The changes in exposure over the areas studied were highly variable, with only relatively small numbers of participants experiencing the larger changes in noise level (7, 12, and 14 dB: L_den_ or similar). However, for the majority of participants the change was much smaller, perhaps 1 to 2 dB. Changes in two of the studies included increased exposures as well as decreased exposures. It would appear that attempts to carefully design a study associated with changed flights paths to ensure a good distribution of changed exposures is difficult because of differences in what is initially proposed (and used as the basis for intervention study design) and what is actually implemented in terms of flight paths and aircraft numbers. The changes at Zurich were particularly related to changes in number of flights in the shoulder hours: early morning and late evening.

In all three studies, there was evidence that the changes in noise exposure, as a consequence of the flight path changes, resulted in change in annoyance outcomes and that these observed changes were statistically significant.

With respect to the strength of association between the change in exposure and the change in annoyance responses, all intervention studies demonstrated that the response was of a magnitude, *at least* as estimated by a steady-state ERF for annoyance. Both the Zurich and Amsterdam studies estimated a site-specific ERF. The Vancouver study made reference to the FICON [11] synthesis. Further, all provide evidence that the change in response was an *excess-response* to the change—both for decreased and for increased exposures in one study, and for increased exposures in the other two. An interesting development in intervention studies was the incorporation (in both the Amsterdam and the Zurich studies) of both level, and change in level, as exposure variables for participants, for modelling the effects of change. Evidence of excess response was tested statistically in the Amsterdam study and presented graphically in the other two studies.

The Amsterdam study provided evidence on the durability of the excess response – it still being present three years after the intervention—though with one unexplained temporary reduction from the fourth panel survey.

In this group of studies, all three studies either adjusted for confounding, or ruled out confounding by design. Military aircraft noise was accounted for by exclusion. Variables included pertained to year of survey, age, sex, ethnicity, home ownership, degree of urbanisation, time of residence, living satisfaction, noise sensitivity, expectations about the airport and the neighbourhood, coping behaviour, dependency on the airport, fear for aircraft crashes, and a negative attitude towards the airport.

### 7.2. Outcome: Sleep Disturbance

#### Summary: Evidence from New/Closed Infrastructure Interventions

See summary of these papers under Section 7.1.2 above.

In both studies (Table 14), there was evidence that the changes in noise exposure as a consequence of the flight path changes resulted in change in sleep disturbance outcomes. In the Amsterdam study, it was also demonstrated that response was in the same direction, and of a magnitude, as estimated by a steady-state ERF for sleep disturbance for Amsterdam derived from before-intervention responses.

Both studies adjusted for confounding including the same variables as described in Table 13 above.

### 7.3. Outcome: Cognitive Development in Children

#### Summary: Evidence from New/Closed Infrastructure Interventions

As in the three aircraft noise studies in Table 13, the intervention in this study (Table 15) involved the rearrangements of flight path resulting from the opening of a new airport and closure of another. The study found various cognitive effects on children (for both the reduction in exposure, and the increase in exposure). Effects disappeared when the old airport closed, emerging after the new airport opened.

In this study, the risk of bias by residual confounding was minimized by study design. This included: ethnicity, parental education, number of family members, parental occupation, and attrition.

## 8. Evidence: Rail Noise

Three studies (Table 16, Table 17 and Table 18) reporting rail traffic noise interventions met the inclusion criteria, all reporting annoyance outcomes at people’s dwellings. Two were conducted in Germany; one in Hong Kong. Two studies involved rail grinding, which can be considered a source intervention, but as one was also associated with an investigation of the effects of informing the community about the noise intervention, it is included below (Table 18) as an education/communication intervention. All were before and after studies, with two having a further after-survey twelve months after the first.

In two of the studies, the changes in the level of exposure as a result of the intervention were minimal, but in one of these there was, additionally, a communication intervention (Type E).

### 8.1. Outcome: Annoyance

#### 8.1.1. Summary: Evidence from Source Intervention

In this one study, there was evidence that the approximately −10 dB changes in noise exposure as a consequence of the source level change resulted in change in annoyance outcomes; that this difference was statistically significant; and that it persisted more than 12 months after the intervention. However, this was a small study, with only one estimate of noise level change reported across all participants together with the means of their annoyance scores.

This study did not adjust for confounding factors.

#### 8.1.2. Summary: Evidence from New/Closed Infrastructure Intervention

While this was new rail infrastructure in Hong Kong, noise from road traffic overwhelmed the train noise for effectively all participants. This study is not reported on further.

#### 8.1.3. Summary: Evidence from Education/Communication Intervention

This study provides some evidence that information communicated to participants about a noise source (as part of an intervention to alter its source levels) has the effect of reducing that community’s response to the noise.

This study did not adjust for confounding but took age, gender, education and coping into account in a descriptive manner.

## 9. Discussion

### 9.1. Overview of Findings Across All Studies

#### 9.1.1. Change in Health Outcomes

Below we provide an overview, across source types, health outcomes, and intervention types, as to whether the intervention resulted in a change in the health outcome, and observations on the magnitude of that change. Table 19 below is a summary overview of the findings reported for each individual study in the sections above. Studies that reported more than one health outcome are entered under both outcomes.

Table 19 shows that most interventions involved road traffic noise (77%), with fewer aircraft noise (16%), and railway noise (7%). The exposure-related interventions in most of the entries in Table 19 were associated with a decrease in environmental noise exposure. However, in five studies (four road traffic noise studies and one aircraft noise study), some or all of the participants experienced noise exposure increases. Observations below with respect to change in responses refer to both the increases and the decreases.

Nearly all of the entries in Table 19, irrespective of the noise source, health outcome, or intervention type, show that the intervention led to a change in the aggregate health outcome of those who experienced the intervention (asterisk shown in the *YES* column). Excluding those studies for which no observation was appropriate (because there was no change in exposure, or the study was a follow-up survey at some interval after the original) there was only one transport noise study reporting no change in health outcomes. The original authors had provided statistically significant tests of this change in 51% of the entries (red asterisks); in a further 37% of entries this observation was interpreted, by the original authors or as part of the process of this review, from the data, tables, or plots presented in the papers, but without statistical tests (black asterisks).

Table 19 also provides an overview of the observed magnitude of change in health outcome as a result of the interventions. Seventeen studies of source, path, and new/closed infrastructure interventions for road and aircraft noise sources reported that the minimum magnitude of the change in outcomes (16 of the studies were of change in annoyance outcome; one of change in sleep disturbance outcome) could have been predicted from a relevant exposure–response function (ERF)—and all but two of these also found there to be an *excess response*—a *change effect* in addition to the *exposure effect* predicted by an ERF. In other words, the reduction in outcome was greater than would be expected based merely on the reduction in noise levels. Brown and van Kamp [15] reported that, for road traffic studies, and source intervention changes, the excess-response change-effect tends to be greater (often much greater) than the change in annoyance due to the noise level exposure change itself. Table 19 shows that observations of excess response in annoyance were for both road traffic (13 studies) and aircraft noise (3 studies).

In general, interventions at the source, in the pathway and intervention in infrastructure (Types A to C) are effective in reducing annoyance, but the available evidence is too poorly conditioned across different group of studies to be able to test for any differences in change in health outcomes arising from different types of interventions.

There is also no clear evidence with respect to thresholds regarding changes in health outcomes as a result of interventions. Intervention thresholds could have two dimensions: (1) the smallest change in exposure levels that result in a change in outcome, and (2) the minimum before-level. The only observation we can make is that several interventions that reduced noise exposures by −1 to −2 dB (energy-based scales) did not result in any observed change in health outcomes.

When interpreting the results, the quality of evidence for various combinations of source, intervention type, and outcome, needs to be considered. The overall quality of evidence within each of the source/intervention type/outcome groups varied, and was judged to range from high to very low across the different groups (see details in Supplementary File 5). It should be noted, however, that for all rows of Table 19 that contain more than two studies, the grouping are assessed as having either high or moderate qualities of evidence (other than sleep disturbance from aircraft noise for new/closed infrastructure which has a moderate quality of evidence).

The influence of contextual, situational, personal factors has to be accounted for. The following factors came forward from the review: noise sensitivity, distance to the road, availability of a quiet side, and window opening behaviour. Additionally, the context around the intervention should be considered, such as attitude towards policy and the party carrying out the measures, expectations about effectiveness of the intervention and satisfaction with the residential area. Only a few studies incorporated these types of factors into their analysis of change in health outcomes.

The studies of ‘other physical interventions’ (such as the provision of a quiet side to the dwelling, or the provision of green space in the neighbourhood) were not intervention studies per se as they did not provide direct evidence of an intervention. Instead, they provide comparisons of health outcomes from groups with and without the particular physical dimension of interest. These ‘other physical interventions’ did, in the majority of studies, demonstrate the efficacy of potential interventions of this sort, but it must be noted that this is indirect evidence consisting of comparison of outcomes of different groups under different conditions, rather than before-after comparisons on the same group.

#### 9.1.2. Sustainability of the Change in Health Outcomes

Nearly all of the entries in Table 19 were before-and-after studies, with the identification of the magnitude of the change in outcome fixed by the timing of the after-survey following the intervention. This was normally one to twelve months after the intervention, but varied considerably. For some of the interventions involving construction, such as barriers or housing reconfiguration, the gap between before and after studies was much longer: five to six years, and eight to ten years, in some studies.

However, a handful of studies continued to assess participant health outcomes longitudinally beyond the initial after-survey. Four road traffic studies, two aircraft studies and two railway studies resurveyed participants after various intervals: five surveys out to 20 months; six surveys out to three years; 12 months; two years, seven to nine years, etc. While the evidence is meagre and scattered, the consistent finding is that the latter after-surveys showed no difference in outcomes to those surveys immediately following the intervention—with no diminution in the magnitude of the effect, including excess response if present. The exception was that the survey seven to nine years after the intervention did show some attenuation in the excess response observed at the first after-survey.

In summary, while there is little evidence regarding longer-term changes in health outcomes subsequent to the initial change following an intervention, none of it suggests adaptation (*adaptation* being defined [54] as movement of the observed excess response, post intervention, towards expected steady-state response levels).

### 9.2. Implications 

#### 9.2.1. Implications for Noise Policy and Management

1. This review has provided a positive answer to an important policy question: *do environmental noise interventions change health outcomes?* This finding is largely consistent across the transport noise interventions. It shows that many current noise management strategies have a beneficial effect on human health. The caveat is that this evidence is not extensive or well distributed over all transport noise sources, intervention types, or health outcomes.
2. Another finding is that relevant ERFs for annoyance can provide an estimate of the *minimum* change in human outcomes that can be expected from a given change in exposure as a result of an intervention. This supports current noise management as ERFs for annoyance can thus provide a first conservative estimate for the health impact assessment of future interventions. The available evidence is more limited for aircraft noise than for road traffic noise. It is also too poorly conditioned across different groups of studies to be able to test for any differences in outcomes arising from different types of interventions. The evidence for ERFs predicting the minimum change in sleep disturbance is restricted to one aircraft noise intervention study only.
3. The review demonstrated that there was *excess response* to the intervention in 14 road traffic noise interventions and three aircraft noise interventions. Excess response occurs where the total difference between the before-outcome and the after-outcomes is greater than the magnitude of the change in response estimated from an ERF for the given change in exposure. A similar result was found for sleep disturbance for one aircraft noise study. The notion of excess response to interventions has been considered in depth by Brown and van Kamp [54] where they examined, and rejected some of, the many explanations that have been proposed for this phenomenon. This study found that: ‘*The evidence of the magnitude, and the persistence over time, of the change effect … and the existence of plausible explanations for it, suggest that it is a real effect and needs to be taken into account in assessing the response of communities in situations where noise levels change. Within the limitations of existing evidence on change, communities that experience an increase in noise exposure are likely to experience greater annoyance than is predicted from existing exposure–response relationships, and communities that experience a decrease in exposure experience greater benefit than predicted. Policy makers need to be informed of these potential* change effects, *particularly as situations in which noise levels increase as a result of infrastructure changes are always likely to be contentious. To do otherwise would be to deny them important information regarding potential community response in these contexts*’.

#### 9.2.2. Guidance for Future Studies of Interventions

The following are implications arising from this review for further research into the health effects of interventions:

1. Further studies directly linking environmental noise interventions to health outcomes are required, for all sources of environmental noise, but particularly for aircraft and rail noise sources, and for human health outcomes other than annoyance.
2. Authorities proposing/funding interventions, whether at local, national, or international level, and whether or not the primary purpose of the intervention concerns noise, should be encouraged to include significant funding for the design and implementation of studies to evaluate outcomes from the interventions. At present, many of the evaluations appear to be addendums to, rather than integral components of, the interventions.
3. The effect of the intervention on the exposure of defined populations needs to be assessed, and its effect on the health outcomes of the same populations – not just the changes in noise levels that result from the intervention.
4. Intervention studies should use validated, and where possible, harmonised, measures of exposures and outcomes, as well as of moderators and confounders.
5. We recognize the difficulty in doing so in many intervention studies, but precise specification of the change in exposure for individuals, or subgroups, is desirable. In part to encourage this, we suggest that there are advantages in following the approach used in two of the individual aircraft noise studies [47,48] of reporting both the noise exposure before the intervention, and change in noise exposure as a result of the intervention, of the study participants, and using both in the analysis.
6. Most interventions result in step changes in exposure with expected step changes in human response to this change in exposure. While many intervention studies use a before and after design, there is generally insufficient consideration that the change in human response to a step change in exposure may have a different time course to that of the change in exposure.
7. A protocol is required for the conduct of future intervention studies that provides longitudinal assessment of both exposure and human response, and Brown [17] reported a design that is suitable (included below as Table 20). With a change in noise exposure over the interval between t_0_ and t_1_, sequential measurements of effect should be made before and after the change, preferably with multiple after measurements (A_−1_, A_0_, A_1_, A_2_, … A_x_). Repeated measurements should also be made of activity interference (Act_x_), potential confounders such as noise sensitivity (Sens_x_), coping strategies (Cop_x_), and a range of other attitudinal, retrospective, and prospective assessments. In addition, that model incorporates steady-state controls into the study design. The protocol in Table 20 is specific to studies of the effect of interventions on annoyance, but the principles of longitudinal measurements of exposure, of responses, and of potential confounders, can be adapted readily to studies of other human outcomes.
8. In reporting the evidence for excess response (in annoyance outcomes) above, we noted that an excess response occurs when the magnitude of the observed change in outcomes is greater than that ‘predicted’ by the ERF, irrespective of whether the observed before and after outcomes themselves lie on the ERF curve. We have noted a tendency, in many studies in which there is evidence of an excess response, for the observed before-outcomes to be much higher than would be indicated by synthesised ERFs. Authors of these individual studies did not explain these higher than anticipated before-responses. We also note the comment by Baughan and Huddart [20] that it is only high noise level situations that receive interventions to reduce noise exposures. In short, intervention studies are biased towards noise situations that are ‘hotspots’. We leave this as an observation only as we have no evidence from this, or previous, reviews as to any mechanism that would lead to changes in reported outcomes from such hotspots (but see a range of potential explanations for excess response in Brown and van Kamp [54] that may have application to ‘hotspots’).
9. We note that the noise exposure metrics reported in the individual studies reviewed did not include a metric that dealt specifically with noise events in transport noise time histories. One exception is the study (Table 20) by Brown (2015) [17]. Participants in that study responded to a noise intervention that focused on a change in the number of noise events, even though there was no change in energy-based noise metrics. We flag this as an issue to be considered in future intervention studies for transport noise.

### 9.3. Systems-Wide Considerations

There is a range of systems-wide matters that additionally should be considered in future evaluations of the health outcomes of transport noise interventions. We note them here, largely without comment, except to indicate that few of these matters were raised within any of the papers examined in the systematic review. However, they are important as they provide, in contrast to existing evidence based on a specific intervention within specific space and time bounds, a systems-wide understanding of transport noise interventions. The latter are likely to be important in comprehensive evaluation of the human health effects of transport noise interventions:

1. Spatial scales of interventions and effects will vary from highly local (e.g., noise barrier on a particular roadway) to regional, national (emission limits for motor vehicles), or international (e.g., emission limits for aircraft).
2. There may be lag times between interventions (e.g., regulations specifying vehicle limits which might take years to implement, or which rely on natural turnover in the vehicle fleet) and measurable effect. There may also be lag times between noise reduction and health consequences, e.g., decreased risk of cardiovascular disease.
3. Some interventions are applied for short periods (e.g., temporary flight path changes) vs. permanent interventions (studies of temporary interventions were excluded from the current review).
4. Interventions may result in unintended displacement outcomes. For example, a traffic restriction intervention that forces traffic into surrounding areas, introduces higher exposures in other areas, even though at the point of application the exposure is reduced. Examples include congestion charging in London [55] and the removal of diesel cars in Rome [56]. In these examples, the reduction in noise levels at one location was accompanied by an increase elsewhere and often in a more deprived area.
5. A related consideration is that there may be subgroup differences in health outcomes from an area-wide intervention (e.g., effects on different socio-economic subgroups) and interventions that redistribute exposures across different areas need to be cognizant of differential socio-economic status of populations in these different areas.
6. There may be effects on human health responses to noise generated by interventions in other fields (e.g., intervention with respect to traffic congestion, or planning interventions that alter urban density).

### 9.4. Publication Bias

It is appropriate to note the possibility that publication bias may have influenced the findings of this review. We have no evidence of this, but it is reasonable to suggest that intervention studies that failed to find a change in human-response outcomes may tend to go unreported compared with those that did find a change.

A potential impediment is that some government and private instrumentalities who initiate noise intervention programs may have little interest in undertaking an evaluation of that intervention once a decision to implement it has been taken—avoiding any possible reputational risk that could be associated with a costly intervention later being shown to have little effect on human health.

## 10. Conclusions

1. An environmental noise intervention framework, showing different types of interventions along the causal path between noise sources and human outcomes, and measurement points along the pathway where changes relevant to human outcomes can be measured, has been used to structure this review. The framework also assists in focussing future studies of the effects of noise intervention strategies.
2. This systematic review of the literature, 1980–2014, found, overall, that there has been a limited number of transport intervention studies published that report observed changes in health outcomes, or observed changes in peoples’ exposures, together with quantitative details on the association between change in exposure and change in human health effects.
  The majority of these were for road traffic noise sources; fewer for aircraft noise and rail traffic noise. The principal change in health outcomes assessed was annoyance, with fewer sleep disturbance, cardiovascular effects, and cognitive development in children.
3. We note that there are many studies in the noise management/control literature of interventions, which report a change in noise emissions or in noise levels, but in the absence of reporting of change in health outcomes or of exposures, these do not elucidate the relationship between interventions and health.
4. The consequence is that there is a restricted evidence base on the health effects of transport noise interventions, with studies spread across 16 different groupings (grouped by source type, health outcome, and intervention type). Only two of these groupings source interventions and path interventions for road traffic for the annoyance outcome have more than three studies.
5. A major difficulty for this review was the diversity between studies, even within those categorised in the same group. This was in terms of study designs method of analyses, exposure levels, and changes in exposure experienced as a result of the interventions. In some studies, the changes in noise exposure were variable across participants (sometimes reported in aggregate) and were not always adequately linked to the corresponding change in outcomes.
6. Because of the diversity, a meta-analysis across studies examining the association between changes in level and changes in outcome was not possible. However, the available evidence did show that transport noise interventions changed the health outcomes reported by those who experience the intervention. This is the case irrespective of the source, the outcome or the intervention type.
7. The *minimum* magnitude of the change in annoyance outcomes because of the interventions can be predicted using a relevant exposure–response function (ERF). Further, in the majority of these studies, the magnitude of the change in response to an intervention exhibited a change effect—an excess response in addition to the level effect predicted using an ERF. This evidence was available for studies of road traffic noise sources (and a small number of aircraft noise studies) and largely only for the annoyance outcome.
8. The available evidence did not allow testing for any differences in change in health outcomes arising from different types of interventions, or for different source types. We also could not make observations regarding thresholds for observable changes in health outcomes, other than that several interventions that reduced noise exposures by −1 to −2 dB did not result in any observed change in health outcomes.
9. While there is little evidence available with respect to the longitudinal path of health outcomes changes following the initial change as a result of an intervention, there is no evidence to suggest the initial change in response is not sustained over at least several years—that is, there is no adaptation.
10. Further studies directly linking transport noise interventions to health outcomes are required, particularly for aircraft and rail noise sources, and for human health outcomes other than annoyance. A protocol has been recommended for the design of future studies.
11. While recognising the difficulty in doing so in many study designs, we suggest that future intervention studies should aim for precise specification of the change in exposure for individuals, or subgroups. There are advantages in following the approach [45,46], of reporting both the noise exposure before the intervention, and change in noise exposure as a result of the intervention, of the study participants, and using both in the analyses.
12. Policy makers need to be informed of the existence of the *change effect* associated with interventions, particularly as situations in which noise levels increase as a result of infrastructure changes are always likely to be contentious. To do otherwise would be to deny them important information regarding potential community response to these changes.
13. The results of the studies available to us regarding other physical interventions were obtained primarily through indirect evidence (comparison of outcomes under different conditions, rather than before-after designs). These have proved useful as a means of estimating the efficacy of such potential interventions, but they need to be supplemented by direct evidence.
14. We note, without evidence, that publication bias may have influenced the findings of this review. We also suggest, again without evidence, that government and private instrumentalities that initiate noise intervention programmes may be inhibited in conducting follow-up evaluations of the intervention through a perception of reputational risk in doing so.
15. The environmental noise intervention studies included in this review focussed on changes at the site of the interventions. We have indicated that there is a range of system-wide factors that also need to be considered in any comprehensive evaluation of the human health effects of any particular environmental noise intervention.

## Figures and Tables

**Table 1:** Categorisation of Noise Interventions.

Type	Intervention Category	Intervention Sub-Category	Examples
A	Source interventions	change in emission levels of sources	motor vehicle emission regulation; rail grinding; road surface change; change in traffic flow on existing roadways/railways; change in number of aircraft flights
		time restrictions on source operations	airport curfew, heavy vehicle curfew
B	Path interventions	change in the path between source and receiver	noise barrier
		path control through insulation of receiver’s dwelling	insulation of building envelope
C ^1^	New/closed infrastructure	opening of a new infrastructure noise source, or closure of an existing one	new flight path; new railway line; new road bypass; or closure of any of these
		planning controls ^2^ between (new) receivers and sources	urban planning control; ‘buffer’ requirements ^2^
D	Other physical interventions	change in other physical dimensions of dwelling/neighbourhood	availability of a quiet side; appearance of the neighbourhood; availability of green space
E	Education/communication interventions	change in behaviour to reduce exposures; avoidance or duration of exposure	Educating people on how to change their exposure
		community education, communication	Informing people to influence their perceptions regarding sources, or explaining reason for noise changes

^1^ Intervention Type C is introduced to categorise situations where noise levels from a source have changed from (say) non-existent to high because of new infrastructure—e.g., from very little road traffic to now being beside a newly opened freeway; or in an area now under a new flight path where previously there had been no overflights. Type C interventions also include the converse: where say road traffic noise drops from a high level because a roadway had been closed, or aircraft noise is eliminated because an airport runway has been shut. Of course, changes in transport infrastructure may produce consequent changes in traffic load on other parts of the network leading to changes (increases or decreases) in source levels, but these are best categorised as Type A Source interventions as they are changes in levels from an existing source. Type C is intended to describe interventions where a (completely) new source is introduced, or an existing one removed—though the distinction will sometimes be blurred [7,8]. ^2^ With Intervention Type C describing opening a new noise source (say, roadway) near an existing dwelling, we extend this category to also incorporate building a new dwelling near an existing noise source. In an urban planning sense, a noise management ‘intervention’ that may be used is the requirement of some minimum distance between existing noise source and new residential development. The effect of such an ‘intervention’ could be measured by comparing human outcomes in newly constructed dwellings at different distances from the same noise source.

**Table 2:** Number of Individual Studies within each Group (Noise Source × Outcome Measure × Intervention Type).

	Number of Peer Reviewed Papers	Number of Non-Peer Reviewed Papers	Total Papers per Group
**ROAD TRAFFIC NOISE SOURCES**			
**Outcome: Annoyance**			
A Source Intervention	7	3	10
B Path Intervention	4	2	6
C New/Closed Infrastructure	1	1	2
D Other Physical	6	1	7
**Outcome: Sleep Disturbance**			
A Source Intervention	1	-	1
B Path Intervention	1	1	2
C New/Closed Infrastructure	2	-	2
D Other Physical	1	-	1
**Outcome: Cardiovascular Effects**			
D Other Physical	4	-	4
Outcome: Modelled Change in Exposure/Effect *			
A Source Intervention	1	1	2
**AIRCRAFT NOISE SOURCES**			
**Outcome: Annoyance**			
B Path Intervention	1	-	1
C New/Closed Infrastructure	2	1	3
**Outcome: Sleep Disturbance**			
C New/Closed Infrastructure	1	1	2
**Outcome: Cognitive Development in Children**			
C New/Closed Infrastructure	1	-	1
Outcome: Modelled Change in Exposure/Effect *			
A Source Intervention	1	-	1
**RAIL NOISE SOURCES**			
**Outcome: Annoyance**			
A Source Intervention	-	1	1
C New/Closed Infrastructure	1	-	1
E Education/Communication	-	1	1

* The modelled outcomes are described in Supplementary File 4.

**Table 3:** Source Interventions (Type A).

Authors	Intervention & Study		*N*, Response Rate & Method	Exposure Levels		Change in Levels and Distribution of Change across Participants	Outcome Measure(s) before and after Outcomes	Did Outcome Change with Change in Exposure? Yes/No (Significance Tested?)	Before/after Outcome Change Compared to That Estimated from an ERF	Comments	Confounders Adjusted for in Analyses
	Nature	Design		Before	After						
Brown (2015) [17]	Brisbane, Truck restriction, change in traffic composition Note: the date of this paper was outside the search time period	B/A. Five rounds of after surveys out to 20 months	99 in panel Response rate 84% ~20% of panel drop out each survey round Interviews	65–73 L_den_ 60–68 L_night_ 69–77 L_10,18 h_ Measured	65–73 L_den_ 60–68 L_night_ 69–77 L_10,18 h_ Measured	No change in L_den_, L_night_ or L_10,18 h_ But see comments All Ps experienced same change—but were exposed to different before levels	%HA based on 7, 8 & 9 of ISO (but with 0–9 scale). B: 58% HA A: 33%, 18%, 18% HA respectively at survey rounds 2, 3 & 4 Mean Annoyance also reported	n.a. as no change in L_den_ exposure (but there was a change in number of noise events) Est. Marg. Mean annoyance scores changed significantly over period of truck restriction (F4,170.4 = 12.18, *p* < 0.001) (see comments)	ERF cited was Miedema & Oudshoorn (2001) [13] 58% HA in before-study much higher than estimated by ERF (latter is 16 to 30% for L_den_ over the range of Ps’ exposures No observation possible on the relationship of change in outcomes with the ERF because L_den_ did not change	Change in response attributed to change in number of noise events	Noise sensitivity; neighborhood quality; respondent association with trucking industry.
Pedersen, Le Ray, Bendtsen & Kragh (2013) [18]	Copenhagen Resurfacing with noise reducing pavement.	B/A study 12 mo. After Not repeated measure	2870 over two areas near roads Response rate 41% Mail surveys	42–74 L_den_ Modelled noise map. Note: wide range of before levels	38–70 L_den_	Measured 4 dB reduction in source levels Same reduction assumed for all Ps	%HA based on 8, 9 & 10 of ISO (0–10 scale) Mean Annoyance also reported	Yes B&A mean annoyance scores were different (Welch’s *t*-test, *p* < 0.001)	Authors reported logistic regression ERFs for each of before and after conditions (*n* = 2870). The 95% CIs of B & A curves tended to overlap, and authors merged the data to establish the ERF. Hence change in response to −4 dB intervention estimated by the ERF. B & A ERFs curves are overlapping—largely parallel but with ERF (after) slightly lower than ERF (before). Response to change estimated by ERF. Slightly lower ERF(A) indicates excess response. The authors also report *‘…a small tendency to a lower %HA in the 50–60 dB range in the after situation…’.*	Merged ERF was higher than Miedema & Oudshoorn (2001) [13] ERF over 60–74 L_den_	
Stansfeld, Haines, Berry & Burr (2009) [19]	UK Bypass roads constructed reducing traffic flow in three small towns	B/A study B:1 year A: 6–7 mos	17 5 exposed 184 control Response rate B:70% A: 74% 67 Ps at exposed area follow-up Delivered questionnaire	L_10,3 h_ (& L_eq,3 h_) Exposed: 75–78 Control: 55–58 Measured Includes train noise	See next column	Change in L_10,3 h_ of −2 to −4 dB suggested for most locations No reporting of distribution of these small changes across Ps.	‘Standard’ noise question for assessing level of annoyance with environmental noise at home. No significant change in mean annoyance score with intervention.	No change in annoyance. Explanation was that the change was too small to be noticed	n.a.	Changes in traffic flow on source roads were small: 24 h flow changed from 26 k to 23 k veh/day, and 24 k to 21 k veh/day	
Baughan & Huddart (1993) [20]	U.K. Decreased traffic flow at 14 sites; increased traffic flow at 6 sites; 2 control sites	B/A study + controls 1–2 mos B&A changes Repeated measure	33–50 per site Response rate and dropout rate not reported Interviews	L_10,18 h_ Decrease sites: 66–76 Increase sites: 65–78		L_10,18 h_ 14 sites with changes ranging from −15 to +5 dB	7 point numerical scale of satisfaction with level of road traffic noise with endpoints labelled *Def. Satis*. And *Def. Unsatis* Outcome reported as mean dissatisfaction score	Yes Infer from next column No statistical tests reported	Authors refer to ERF derived from ‘TRRL’ survey at 35 steady-state sites. Authors conclude: For decreases, both before and after levels (of dissatisfaction) differed significantly from steady state (ERF). B/A transitions steeper than ERF; For increases, after levels differed significantly from steady state. B/A transitions steeper than ERF; No statistical tests reported; Response to change in same direction as estimated by ERF, but much steeper, indicating excess response	Data used in Griffiths & Raw (1989) [21] below also included in analysis in this paper	
Griffiths & Raw (1989) [21]	England. Repeated measure of after survey in Langdon & Griffiths (1982) [22] 5 sites	Repeat of After study at 7–9 years. After 48% of Ps repeat interview	430 Interviews	See Langdon & Griffiths (1982) [22]			Four-point verbal bother scale Outcome reported as mean bother score for each of B&A conditions	n.a.(because there was no change in exposure between 7 and 9 years)	See results in Langdon & Griffiths (1982) [22] below. Observed Excess responses show no diminution out to 2 years after change, but is diminished, but still exists, 7–9 years after the change		
Brown (1987) [23]	Brisbane. Increase in traffic flow	B/A study 2 weeks B, 7 & 19 mos A Repeated measure	20 Response rate 83% Interviews	L_Aeq,24 h_ 60 L_10,18 h_ 60 L_dn_ 61	L_Aeq,24 h_ 66/67 L_10,18 h_ 68/71 L_dn_ 69/71	L_Aeq,24 h_ + 6/+7 dB L_10,18 h_ +8/+11 dB L_dn_ +8/+10 dB	7 point semantically labelled annoyance scale. Reported individual responses and %HA based on top two categories.	Yes Distribution of individual annoyance responses changed after intervention (Friedman Two-way Anova, *p* < 0.01). 90% CIs for %HA B & A intervention do not overlap	ERF cited was Schultz (1978) [10] and plotted as band containing 90% of data points used in Schultz synthesis. Before %HA lay within Schultz 90% band, After %HA lay above ERF (though Cis for %HA are wide due to small sample size). Indicates excess response to increase in exposure	Note: No evidence of adaptation. Distribution of annoyance scores not different at 7 and 19 mos after change (*t*-test, *p* < 0.05)	
Griffiths & Raw (1986) [24]	England. Decreased traffic flow at 6 sites; increased traffic flow at 2 sites	B/A study 1–4 mos before change 2–3 mos after change Repeated measure	469 Response rate 74% 17% drop out between surveys (391)	L_10,18 h_ Decrease sites: 65–81 Increase sites: 54–56 Measured and calculated	L_10,18 h_ Decrease sites: 66–74 Increase sites: 61–69	Change in L_10,18 h_ at seven sites were: (1) −14.5 dB (2) −5.7 dB (3) −2.6 dB (4) −3.1 dB (5) −1.3 dB (6) +5 dB (7) +15 dB	7 point numerical scale of satisfaction with level of road traffic noise with endpoints labelled *Def. Satis.* And *Def. Unsatis* Reported site mean dissatisfaction scores	Yes Infer difference between B&A mean dissatisfaction scores from next column	Authors calculated ERF using ‘steady-state’ before responses. Site mean dissatisfaction scores regressed against before L_10,18 h_. Mean dissatisfaction scores (After) were compared to those estimated by the ERF. For decreases: decrease in site mean dissatisfaction score was greater than estimated by a conservatively estimated ERF (*t =* 3.14, df = 4, *p* < 0.025). Similarly, at two increase sites, increase in individual dissatisfaction score was greater than estimated (*t =* 2.93, df = 81, *p* < 0.005). Response to decrease/increase changes in direction estimated by ERF, but steeper—hence excess response	Note: resurvey of three decrease-sites out to 17–22 mos. After change showed no change in observed excess response. Griffiths & Raw (1989) [21]	
Brown, Hall & Kyle-Little (1985) [25]	Brisbane. Reduction in traffic flow	B/A study with 2 control groups (quasi experimental)	49 Cntrls: 52, 40	L_10,12 h_ 74.3 Calculated Cntrl. 75.1 Measured	L_10,12 h_ 64.5 Measured Cntrl. 65.2 Measured	L_10,12 h_ −9.8 dB	7 point semantically labelled annoyance scale. Reported %HA based on top two categories. Annoyance with before conditions assessed in retrospect.	Yes Note, before %HA based on retrospective assessment No statistical test reported—but see next column	ERF cited was Schultz [10] and plotted as band containing 90% of data points used in Schultz synthesis. After %HA and Control sites %HA lay within 90% band. Before %HA (retrospective) lay outside 90% band. Response to decrease in same direction as estimated by ERF, but steeper, indicating excess response	This study relied on retrospective assessment of annoyance before the change	
Langdon & Griffiths (1982) [22]	U.K. Reduction in traffic volumes at 6 sites after opening of new relief roads	B/A study 2–3 mos. B to 4–6 mos. A	Number of respondents at each of the six sites not reported	L_Aeq,24 h_ 72–76.5	L_Aeq,24 h_ 56.5–73.5	Change in L_Aeq,24 h_ 6 sites ranging from −5 to −15.5 dB All Ps at a site experienced the same change in exposure	Four-point verbal bother scale Outcome reported as median of bother score for each of B&A conditions at all six change sites	Yes Infer difference between B&A median bother scores from next column	Authors compare change data to ERF from eight sites in London where exposure and response were measured under steady-state condition. For the six change sites: No sig. diff. between median Before bother scores and scores estimated by ERF (*t*-test = 2.13, *p* > 0.05). Sig. diff between median After bother scores and scores estimated by ERF (*t*-test = 8.25, *p* < 0.001) Response to change in same direction as estimated by ERF, but steeper, indicating excess response		
Kastka (1981) [26]	Germany Complex set of noise and traffic control measures in 6 cities Plots 50 data points	B/A study	1800	Measured L_d_ (range 47–68 B, 50–65 A		Range of sites with changes of −8 to +3 dB (mean −1 dB)	Complex set of measures including assessment of sensory experience and interferences of noise	Yes Infer from next column No statistical tests reported Percentage highly annoyed in line with an extra 6 dB from steady-state scale	Author reports ERFs of both response measures on L_D_ for both B and A conditions After ERFs much lower, but somewhat parallel to the Before ERFs Shows strong excess response, equivalent to that of a 6 dB (8 dB for the second response measure) change in exposure No statistical test reported	This study has been included under Type A source interventions even though it is not fully clear exactly what intervention(s) were responsible for the change in response	

Abbreviations used in this and all subsequent tables: N: number of participants; B: Before-study; ERF: Exposure response function; n.a.: not applicable or not available; A: After-study; P(s): Participant(s); CI: confidence interval; ISO: ISO annoyance scales (ISO_TS_15666_2003); mos: months; Q: questionnaire; %HA: Percentage Highly Annoyed; s.d.: standard deviation; B/A: Before and After study; SE: standard error.

**Table 4:** Path Interventions (Type B).

Authors	Intervention & Study		*N*, Response Rate & Method	Exposure Levels		Change in Levels and Distribution of Change across Participants	Outcome Measure(s) before and after Outcomes	Did Outcome Change with Change in Exposure? Yes/No (Significance Tested?)	Before/after Outcome Change Compared to That Estimated from an ERF	Comments	Confounders Adjusted for in Analyses
	Nature	Design		Before	After						
Amundsen, Klaeboe & Aasvang (2011) [27]	Norway Façade insulation	Two survey rounds: B&A Target; Control & Supplement groups. B & A surveys approx 6 mos. either side of intervention	Target: B: 168 Response rates 57% A: 161 (65%) Control: B: 469 (57%) A: 254 (65%) Supplement: 112 Mail survey	L_Aeq,24 h_ 61–78 outside. Calculated Mean 71 (Av inside level before change is 43)	Façade insulation reduces inside levels by 7 dB	–7 dB for indoor noise levels for all Ps in target group	Standard ISO annoyance scale (5 point verbal). %HA calculated using top two points of scale B: 42%HA A: 16%HA Control: B: 24%HA A: 29%HA	Yes Intervention resulted in substantial and significant (*p* < 0.001) reductions in individual annoyance scores	Authors chose not to compare their results with Miedema & Oudshoorn ERF [13]. Fitted a model of individual annoyance responses to outdoor levels for all Ps (target, control and supplementary: *n* = 738) with *receiving the intervention* as a dummy variable. Estimate of effect size −0.820 (*p* < 0.000) and 95% CI −1.170 to −0.470. Authors claim size of annoyance reductions with intervention is in line with ERF modelled from individual indoor levels. However this appears to be contradicted by the large reduction in the Target Group’s %HA (42% before intervention to 16% after). Summarised as ‘unclear’	Authors note no explanation why %HA sig. lower in control than target before intervention; and second round higher than first in control	Gender, age, education level, marital status, access to a bedroom on the quiet side of the building, and sensitivity
Amundsen, Klaeboe & Aasvang (2013) [28]	See Amundsen, Klaeboe & Aasvang (2011) [27] above. Same study details but this was a repeat survey 2 year after first post-intervention study. Mailed to all Ps who had completed first post-intervention study. Number of participants now 104 (Response rate 58%) in target; 139 in Control; 63 in supplement						2nd after-study: A: 16%HA	Result the same two years after initial After survey (*p* < 0.01) Additionally, repeated ANOVA was conducted on panel who had answered all three survey rounds (*N* = 212). Change in annoyance as a result of intervention significant in first (*p* < 0.0005) but not second (*p* = 0.33) after survey	In this repeated ANOVA, multivariate partial eta square = 0.44		
Bendtsen, Michelsen & Christensen (2011) [29]	Denmark Enlargement of motorway lanes but with dwelling insulation, barriers, & quiet pavement	B/A study 1 year before constr & 1 year after B/A gap 6 year	Q sent to 1200 dwel. In 6 areas out to800 m from motorway Response rates B:71% A: 65% 38% B&A Mail survey	L_den_ 45–65 Calculated. Unclear as to whether calculated levels included traffic sources other than motorway	L_den_ 45–60 Calculated. Not reported is whether some Ps may have experience increased after-levels	Reductions in extent of exposure 60–65 & 55–60 bands but increase in lower two bands. Reported only at population level. No indication of the change experienced by individual Ps	ISO scale (5 point verbal) % top three annoyance categories dropped, other two categories increased Top two categories (%HA—but authors did not use this term) dropped from 37% to 16%	Yes but no data presented of change in exposure of those reporting change in annoyance No statistical tests	n.a.	Classed as path intervention, even though includes quiet pavement as intervention Multiple sources of road traffic exposure—not just motorway	
Gidlöf-Gunnarsson, Öhrström & Kihlman (2010) [30]	Sweden Full-scale filling-in building gaps; barriers & housing improvement	B/A study 5 year apart	B: 160 Response rate: 56% A: 153 (47%) Mail survey	L_Aeq,24 h_ at façade 48–71 Calculated		–5 to −10 on exposed facades; −4 to −10 courtyards	ISO scale (5 point verbal) %Annoyed cut-off includes top 3 points.(Note: NOT %HA) For Ps highly exposed and with large change: B: 84% Annoyed A: 28% Annoyed For P’s with less change: B: 45–55% Annoyed A: 21–22% Annoyed	Yes Large and consistent reductions in %Annoyed associated with reduction in noise exposure (but no statistical tests)	Authors refer to Öhrström [7] who cites ERF of Miedema & Vos [12] For Ps in most exposed part of study, B/A 84/28%Annoyed outcomes both higher than estimated by this ERF, but also show much larger decrease in response than estimated by ERF. Response to change in same direction as estimated by ERF, but steeper, indicating excess response. (But no statistical tests)	This was a reconstruction project that included many other environmental changes—not just change in noise exposure (Ps reported 36% increase in overall satisfaction with area)	
Kastka, Buchta, Ritterstaedt, Paulsen & Mau (1995) [31]	Germany Noise barriers at 12 sites + 2 control sites	B/A study 1–2 years B, 8–10 years A barriers were built	283 B Response rate 59% 212 A (72%) 97 Ps both B&A	L_eq,D_ B 50–70 Measured	L_eq,D_ A:51–66 Measured	–13 dB close to barriers to 0 dB at 200 m Av. Change −4.1 dB	(1) 5 point verbally labelled disturbance scale (2) %HA calc. as top two responses on scale in (1) (3) factor K1: sensory-perceptional and emotional experience of traffic noise (0–10) (4) factor K2: noise interferences	Yes All response variables show significant reductions, e.g., %HA B: 64%; A:35% (chi^2^ = 39.69 *p* < 0.005) Control sites response variables same B&A	Authors calculated an ERF using the steady-state before-responses. For this, mean disturbance scores (and, separately, other outcome variables including %HA) were regressed against before L_eq,D_ Mean (After) disturbance scores were compared to those estimated by this ERF. At 11 of the 12 sites, estimated mean disturbance score was greater than observed. Difference was statistically significant (matched pair *t*-test, df = 11, *p* < 0.05). Response to change in same direction as estimated by ERF, but steeper, indicating excess response following barrier construction	Authors reported extensive additional analyses They suggest no simple causal relation between noise level reduction and annoyance reduction	
Nilsson & Berglund (2006) [32]	Sweden Noise barrier	B/A study + control 9 mos. B; 15 mos. A Repeated measures on 59%, 46% only	Before 304 Response rate 77% (241 control Response rate 66%) (After Response rates: 72%, 69%) Self-administered	L_den_ 70 to <45 Calculated	L_den_ 62.5 to <45 Calculated	–7.5 dB; with reducing change out to 100 m from barrier. Distribtn of change was: –7.5 dB 52 Ps −5 dB 47 Ps −2.5 dB 31 Ps	Visual analogue scale 7-point annoyance scale. Transformed to 0–100 scale. Reports %HA as above cut-off 72	Yes Reductions in %HA were significant (*p* < 0.05, sign-test) for three groups of Ps within 100 m of roadway Control: no diff in B&A %HA	ERF cited was Miedema & Oudshoorn (2001) [13] Reports both B&A %HA agree with prediction by ERF (no statistical test) Response to change same direction and magnitude as estimated by ERF	Outdoor annoyance did not conform to ERF	
Vincent & Champelovier (1993) [33]	France Noise barriers and low noise road surface	B/A study at 2(?) sites.	75 Response rate not reported	L_eq,12 h_ 65.1 Location of measurement site relative to Ps not reported	L_eq,12 h_ 56.3 Location not reported	Change in levels was variable with distance from road: −10 to −3 dB between 10 and 100 m.	% highly annoyed (scale and definition of HA not reported). B: 22%HA A: 8%HA	Yes (but no statistical test)	No comparison of change to any ERF	Author notes that response to ‘Often disturbs sleep’ dropped from 13% to 6%	

**Table 5:** New/Closed Infrastructure (Type C).

Authors	Intervention & Study		*N*, Response Rate & Method	Exposure Levels		Change in Levels and Distribution of Change across Participants	Outcome Measure(s) before and after Outcomes	Did Outcome Change with Change in Exposure? Yes/No (Significance Tested?)	Before/after Outcome Change Compared to That Estimated from an ERF	Comments	Confounders Adjusted for in Analyses
	Nature	Design		Before	After						
Gidlöf-Gunnarsson, Svensson, & Öhrström (2013) [8]	Stockholm Opening urban road tunnel reduced traffic on road system	B&A Exposure and control groups 1 year B & 1 year A Repeated measures	Exposure group: B:758 Response rate 55% A: 493 (75%) Control: B: 311 A: 165 Analysis based on 658 in both B&A Mail survey	L_Aeq,24 h_, 48–71 Control: 52–66 Measured/some estimated	See next column	194 Ps: −11 to −17 dB 225 Ps: –3 to −5 dB Control: no change in levels	ISO scale (5 point verbal) %Annoyed (note: not %HA) calculated using top three points of scale Exposure group: B: 60% Annoyed A: 20% Annoyed Control: B: 24% Annoyed A: 29% Annoyed	Yes Intervention resulted in substantial and significant (McNemar-test, *p* < 0.001) reduction in annoyance over the exposure area—but no change in control area	Authors cite Miedema & Oudshoorn (2001) ERF [13]—but refer only to %Annoyed, not %HA (uses L_den_ = L_Aeq,24 h_ + 4) Authors also fitted a model of individual annoyance responses to exposure levels for all Ps, but using the exposure levels AFTER the intervention (*n* = 437: excluding Ps in one study sub area and control). Authors report that these modelled outcomes fit ERF for %Annoyed in Miedema & Oudshoorn (2001) [13] However, %Annoyed with exposures BEFORE the intervention was very much higher than indicated by ERF. Thus response to change in same direction as estimated by ERF, but steeper, indicating excess response	Authors suggest their modelling of %Annoyed on after-levels indicates no change-effect. They noted, but did not investigate, the excess response in the overall before-to-after change Authors reported ‘dramatic’ improvement in living environment for Ps with largest noise reduction (note: traffic on nearest road dropped from 60,000 veh/day to zero)	
Öhrström (2004) [7] Öhrström & Skånberg (2000) [34]	Gothenburg Major traffic reduction by construction of tunnel + narrowing of surface roadway	B/A study + control 1 year. B&A tunnel opening. Repeated measure	50 (92 control) Response rate 62% ~15% between surveys Delivered survey forms	67 (range 56–69) Control Av. 45 Calculated Note range of before levels	Av. L_Aeq,24 h_ 55 (range 44–57) Control Av. 44 Calculated	–12 dB Av L_Aeq,24 h_ reduction Distribution of magnitude of the change across individual Ps not reported	%HA based on top category of 4 point verbal scale B: 58%HA A: 7%HA Control B&A 1.1%HA to 0%HA Mean Annoyance on ISO also reported (B: 8.9; A: 1.4)	Yes Sig. diff. (*p* < 0.001) in B&A %HA Sig. diff. (paired *t*-test, *p* < 0.001) in B&A mean annoyance scores	Author refers to ERF of Miedema & Vos (1998) [12]. This ERF indicated %HA should move from approx. 30%HA to approx. 10%HA for the change in exposure experienced in this study. Observed percentages were 58%HA to 7%HA measured in the study group Thus response to change much steeper than ERF indicating large excess response	Note: author claimed no excess response—based on after levels Author speculates large change in response may also be related to air quality, vibration and appearance changes	

**Table 6:** Other Physical Intervention (Type D).

Authors	Study		*N*, Response Rate & Method	Exposure Levels	Outcome Measure(s)	Did Outcome Change with ‘Intervention’? Yes/No Strength of Effect	Comments	Confounders Adjusted for in Analyses
	Nature	Design						
de Kluizenaar et al. (2013) [35]	Questionnaire survey	Cross- sectional Stratified sample on age and district	1967 50% RR	For each dwelling, exposure levels were calculated at the most and least exposed façade (L_den_,_most_ and _Lden,least_, respectively). 40–70 dB	Annoyance ISO scale (0–10 point verbal)	Yes Stronger association between noise and annoyance for those: who have relative quiet available (>10 dB difference between most and least exposed façades). Beta = 0.099 SE = 0.012, *p* ≤ 0.0001, who have higher noise level at the least exposed façade. Beta = 0.035, SE = 0.016, *p* ≤ 0.05) No interaction was confirmed		Age, gender, education, and annoyance from neighbour noise and ‘humming’ noise
Babisch et al. (2012) [36]	(HYENA) study is a large-scale multi-centered study carried out simultaneously in 6 European countries Prevalence of (designed as a hypertension study with air and road traffic sources) Study examined many modifiers. Here only the result wrt quiet side and living room facing the street are reported	Cross-sectional in stratified random samples around 6 airports (but response to road traffic noise examined here)	4861 (45–70 years old) 30–78% RR	L_Aeq24 h_ 45–65 road traffic noise	Annoyance ISO scale (0–10 point verbal)	Yes Location of the bedroom resulted in decreased annoyance at night (Beta = 1.25, CI = 1.12–1.38 vs. Beta = 0.81, CI = 0.65–0.97; interaction *p* < 0.001). per 10 dB Those with location of the living room facing the street were more annoyed during daytime with increasing road traffic noise level (Beta = 1.63, CI = 1.50–1.76) than those whose living room was located on the back side (Beta = 1.44, CI = 1.18–1.69); interaction p = 0.007	Samples based on air traffic noise but models adjusted for this	Full models, both continuous noise levels (Air and Road), type of housing, location of rooms shielding due to obstacles, visibility of the postal street, window opening habits, type of windows length of residence, time spent in the living room on workdays, time spent in the bedroom on workdays noise reducing remedies, building modifications to reduce noise, self reported hearing problems, rooms per occupant
van Renteghem & Botteldooren (2012) [37]	Belgium Effect of presence of a quiet façade on annoyance in high noise exposure dwellings	Comparison: of responses in dwellings with and without a quiet side All dwellings had noisy side: half also had a quiet side	100 Response rate 70% Interviews	L_den_. 65–75 at most exposed façade—all dwellings. Half of dwellings also had quiet side Both levels sourced from END maps	ISO scale (5 point verbal) Analysis used mid category cut-off ‘at least moderately annoyed’	Yes Absence of quiet façade results in increased ‘at least moderately annoyed’ respondents: Odds ratio 3.3 when adjusted for noise sensitivity (95% CI 1.35–8.01) When people actually used the bedroom at the quiet side OR = 10.6.(95% CI 2.0–56)	Quiet side defined as a front/back façade level difference >10 dB	Noise sensitivity, window closing, bedroom on a quiet side,, front-façade Lden
de Kluizenaar et al. (2011) [38]	Questionnaire survey	Data drawn from a prospective cohort study For a postal questionnaire survey	18 973 (15–74 years) 70% RR	For each dwelling, exposure levels were calculated at the most and least exposed façade (L_den,most_ and L_den,least_, respectively). 40–70 dBA (Estimates available for *N* = 17,650)	Total Annoyance Dichotomous scale	Yes Stronger association between noise and annoyance for those who have relative quiet available (>10 dB difference between most and least exposed façade) for all levels >45 dB Ors range: 1.33–6.54 (all significant) Interaction term significant for two noise categories: OR = 3.177 for Lden interval 57.5–62.5; OR = 5.584 for Lden >60		Age, sex, body mass index, exercise, marital status, work situation, financial difficulties, alcohol use, education
Gidlöf-Gunnarson & Öhrström (2010) [30,39]	Sweden Effect of appearance of quiet side courtyard on annoyance in dwelling with high noise exposure	Comparison: of responses in dwellings with and without an attractive courtyard All dwellings had noisy side and a quiet side	385 Response rate 59% Mail survey	L_Aeq,24 h_ Calculated levels Noisy façade in two categories: 58–62 dB (*n* = 241) and 63–68 dB (*n* = 144). All had access to a ‘quiet side’ 239 Ps had low quality courtyard, 146 had high quality courtyard	ISO (5-point verbal) scale Analysis used mid category cut-off *at least moderately annoyed* Percentage of noise annoyed residents was significantly lower across the two sound level categories among those who had high (16% and 29%) than low-quality quiet courtyards (27% and 42%)	Yes Percentage annoyed depended on noisy façade exposure level, but was less when quality of courtyard was high, rather than low Odds Ratio for courtyard quality was 0.59 (95% CI: 0.36–0.96)	Quiet side defined as L_Aeq,24 h_ < 48 including façade reflection Quality of courtyard was assessed objectively.	Type of housing; Lay out and population characteristics: were comparable in the two study groups
Gidlöf-Gunnarsson, & Öhrström (2007) [40]	Sweden Nearby green area	Green versus non green Quiet site available versus not available All areas above 60 dB Most aspects kept constant at similar noise exposures, road traffic dominating source	500 Response Rate 59% Interviews	>60 dB	ISO scale (0–10)	Yes Significant associations emerged for availability to green areas (*p* < 0.001) and for access to a quiet side (*p* = 0.001), However, the effect sizes were low (partial η^2^ = 0.029 and 0.023, respectively)	Interaction quiet side and green space not tested	Age

**Table 7:** Source Interventions (Type A).

Authors	Intervention & Study		*N*, Response Rate & Method	Exposure Levels		Change in Levels and Distribution of Change across Participants	Outcome Measure(s) before and after outcomes	Did Outcome Change with Change in Exposure? Yes/No (Significance Tested?)	Do before and after Outcomes fit with Relevant ERF	Comments	Confounders Adjusted for in Analyses
	Nature	Design		Before	After						
Stansfeld, Haines, Berry & Burr (2009) [19]	UK Bypass roads constructed reducing traffic flow in three small towns	B/A study B: 1 year A: 6–7 mos	175 exposed 184 control Response rates B: 70% A: 74% 67 Ps at exposed area follow-up Delivered questionnaire	L_10,3 h_ (& L_eq,3 h_) Exposed: 75–78 Control: 55–58 Measured some train noise	See next column	Change in L_10,3 h_ of −2 to −4 dB suggested for most locations No reporting of distribution of these small changes across Ps	Jenkins Sleep Scale No significant change in sleep total score	No change in sleep disturbance Explanation was that the change was too small to be noticed	n.a.	Change in traffic flow on source roadways were small: 24 h flow changed from 26 k to 23 k veh/day, and 24 k to 21 k veh/day	SES

**Table 8:** Path Interventions (Type B).

Authors	Intervention & Study		*N*, Response Rate & Method	Exposure Levels		Change in Levels and Distribution of Change across Participants	Outcome Measure(s) before and after Outcomes	Did Outcome Change with Change in Exposure? YES/NO (Significance Tested?)	Before/after Outcome Change Compared to that Estimated from an ERF	Comments	Confounders Adjusted for in Analyses
	Nature	Design		Before	After						
Amundsen, Klaeboe & Aasvang (2013) [28]	See Table 4					L_Aeq,24 h_, –7 dB for indoor noise levels for all Ps in target group	Several sleep questions, but ‘*sleep disturbed*’ based on Yes/No response to either of: ‘*I am disturbed by traffic noise*’ or ‘*I wake up because of traffic noise*’ B: 45% disturbed A: 22% disturbed	YES %Sleep Disturbed dropped after intervention (*p* < 0.0005 McNemar’s test) No change in control group Results stayed the same two years after	n.a.	Overall sleep quality also assessed (top two points of 5-point sleep quality scale = ‘*poor sleep*’ Intervention resulted in less ‘*poor sleep*’ similar to change in %Sleep Disturbed	Gender, age, education level, marital status, access to a bedroom on the quiet side of the building, and noise sensitivity
Bendtsen, Michelsen & Christensen (2011) [29]	See Table 4					L_den_ Reductions in extent of exposure 60–65 & 55–60 bands but increase in lower two bands	Unclear. Appears to be based on binary response to two questions: ‘*difficulties in falling asleep*’ & ‘*wake up at night’*	Yes Ps. Reported sleep disturbance (both questions) dropped B: 14 & A: 7% No statistical tests	n.a.	No data presented of change in exposure of those reporting change in sleep	

**Table 9:** New/Closed Infrastructure (Type C).

Authors	Intervention & Study		*N*, Response Rate & Method	Exposure Levels		Change in Levels and Distribution of Change across Participants	Outcome Measure(s) Before and after Outcomes	Did Outcome Change with Change in Exposure? Yes/No (Significance Tested?)	Before/after Outcome Change Compared to that Estimated from an ERF	Comments	Confounders Adjusted for in Analyses
	Nature	Design		Before	After						
Öhrström (2004) [7] Öhrström & Skånberg (2000) [34]	Gothenburg Major traffic reduction by construction of tunnel + narrowing of surface roadway	B/A study + control 1 year. B&A tunnel opening. Repeated measure	50 (92 control) Response rate 62% ~15% between surveys Delivered survey	Av. L_Aeq,24 h_ 67 (range 56–69) Control Av. 45 Calculated Note range of before levels	Av. L_Aeq,24 h_ 55 (range 44–57) Control Av. 44 Calculated	12 dB Av L_Aeq,24 h_ reduction Distribution of magnitude of the change across individual Ps not reported	15 questions on sleep and sleep environment. Ps asked to compare sleep and sleep behaviour with how it was one year earlier—before intervention	YES Sig. diff. (*p* < 0.01) in % exposed Ps reporting improvement, compared to control, in following: sleep with open windows time for falling asleep wakes up sleep quality tiredness in morning	n.a.		
Öhrström & Skanberg (2004) [41]	See row above: Öhrström (2004) [7] Öhrström& Skånberg (2000) [34] Substudy of above. Exposed area 25–67 m from roadway (11 Ps); control area 125–405 m from roadway (13 Ps) Longitudinal study: B & two A: 5 mos and 17 mos after intervention					L _Aeq,24 h_ outside Exposed 11 Ps –10 to −13 dB Control 13 Ps Most 0 to −1 (one P each −4 and −5)	Sleep questionnaire & wrist actigraphy After outcome: Questionnaire: reduced difficulty falling asleep & better sleep quality Actigraphy: fewer long wake episodes & shorter sleep times	Yes Questionnaire & actigraphy showed Ps significant reduction of time in bed (increased sleep efficiency) (*p* = 0.02); increase in subjective sleep quality and less time needed to fall asleep	n.a.	Primary purpose was to test if there was a difference between sleep questionnaire and sleep actigraphy	

**Table 10:** Other Physical Intervention (Type D).

Authors	Study		*N*, Response Rate & Method	Exposure Levels	Outcome Measure(s)	Did Outcome Change with ‘Intervention’? Yes/No	Before/after Outcome Change Compared to that Estimated from an ERF	Comments	Confounders Adjusted for in Analyses
	Nature	Design							
van Renteghem & Botteldooren (2012) [37]	Belgium Effect of presence of a quiet façade on sleep in dwellings with high noise exposure	Comparison: of responses in dwellings with and without a quiet side All dwellings had noisy side: half also had a quiet side	100 Response rate 70% Interviews	L_den_. 65–75 at most exposed façade All dwellings Half of dwellings also had quiet side Both levels sourced from END maps	I A Na: sleep indicators: difficulties in falling asleep, awakening due to noise and window open (4 point scale: *never, sometimes, a lot, always*)	Yes Absence of quiet façade results in increased ‘*at least sometimes*’ respondents: Odds ratio for falling asleep 5.5 (95% CI 0.7–44.1)	n.a.	Quiet side defined as a front/back façade level difference >10 dB	Noise sensitivity, window closing, bedroom on a quiet side, front-façade L_den_

**Table 11:** Other Physical Intervention (Type D).

Authors	Study		*N*, Response Rate & Method	Exposure Levels	Outcome Measure(s)	Did outcome Change with ‘Intervention’? Yes/No Strength of Effect	Comments	Confounders Adjusted for in Analyses
	Nature	Design						
Babisch, Wölke, Heinrich & Straff (2014) [42,43]	Germany Effect of quiet side and type of road on blood pressure	Major and secondary roads, quiet side available or not	1770 (Major road 753, Side street 1017) Response Rate not reported	L_den_ Major road: mean 67 s.d. 7.2 Side street: mean 49 s.d. 4.7	Self-reported hypertension.	11% increase of the risk of hypertension per increment of 10 dB(A) of the road traffic noise level was found Yes 31% higher risk of hypertension along major roads compared to those who lived in side streets In people that lived on major roads, an odds ratio of OR = 1.736 (95% CI = 1.005–2.997, *p* = 0.048) was found for the extreme comparison between both rooms on the front or the rear side of the house	Location of living room more important than location of the bedroom (not in line with other studies)	Age, gender, education, body mass index, physical activity at leisure, alcohol intake, family history of hypertension and occupants per room
Babish et al. (2012) [36]	(HYENA) study was a large-scale multi-centered study carried out simultaneously in 6 European countries Prevalence of (designed as a hypertension study with air and road traffic sources). Study examined many modifiers. Here only the result wrt quiet side and living room facing the street are reported	Cross-sectional in stratified random samples around 6 airports	4861 (45–70 years old) 30–78% RR	L_Aeq24 h_ 45–65 road traffic noise	Hypertension based on blood pressure measurements during home visits (defined as: a systolic BP ≥ 140 or a diastolic BP ≥ 90)	No Location of the bedroom did not result in significantly increased or decreased hypertension (OR = 1.09, 95% CI = 0.98–1.22 vs. OR = 1.10, 95% CI = 0.94–1.28; interaction *p* = 0.555) Location of the living room facing the street did not show an increase in the risk of hypertension with increasing road traffic noise level (OR = 1.06, 95% CI = 0.96–1.17)	Samples based on air traffic noise but models adjusted for this	Full models, both continuous noise levels (Air and Road) type of housing location of rooms, shielding due to obstacles, visibility of the postal street, window opening habits, type of windows length of residence, time spent in the living room on workdays, time spent in the bedroom on workdays noise reducing remedies, building modifications to reduce the noise, self-reported hearing problems, rooms per occupant
Lercher et al. (2011) [44]	Oral and telephone interviews by means of a structured questionnaire	Cross sectional	1653 first wave, 252 second wave 35% & 41% RR	L_den_ 30–78. Calculated.	Self-reported hypertension	No Results show that participants with bedrooms facing toward a quiet yard reveal a clear trend, but non-significant, toward a reduction in hypertension diagnoses in the ALPNAP-study (OR = 0.78, 95% CI = 0.59–1.05).		Age, sex, BMI, family history, education, health status, duration of living, age
Bluhm et al. (2007) [45]	Questionnaire survey	Cross-sectional	667 77% RR	Estimated noise levels dB(A)) annual mean L_Aeq24 h_. Individuals were classified into exposure categories of 5 dBA, from 45 dB(A) to 0.65 dB(A)	Self-reported hypertension	Yes Stronger association between noise and hypertension for those whose bedroom windows was facing the street (OR 1.82; 95% CI 1.22 to 2.70). Also a stronger effect for those who did not have triple glazed windows (OR 1.66; 95% CI 1.17 to 2.34)	Note: The effect of window glazing is ‘indirect evidence’ for a path effect.	Age, type of residence, occupation, smoking (others included but not significant)

**Table 12:** Path Interventions (Type B).

Authors	Intervention & Study		*N*, Response Rate & Method	Exposure Levels		Change in Levels	Outcome Measure(s) before and after Outcomes	Did Outcome Change with Change in Exposure? Yes/No (Significance Tested?)	Before/after Outcome Change Compared to That Estimated from an ERF	Comments	Confounders Adjusted for in Analyses
	Nature	Design		Before	After						
Asensio, Recuero, & Pavón (2014) [46]	Spain 5 airports Window insulation as part of NIP (Noise Insulation Program)	After study only—following insulation. Time since intervention not reported Before by retrospective assessment	689 Random selection from buildings that had been insulated Response rate not reported Telephone interviews	L_day_ > 65 L_night_ > 55 Calculated Actual exposures not reported	Not reported	Not reported	ISO annoyance scale (0–10) Before annoyance asked in retrospect during after-survey, followed immediately by after-annoyance question Annoyance for Day, for Night, & outdoors in neighbourhood were separate questions. Mean annoyance scores for each of these were 8.5, 7.6 and 9.0	Yes Mean Day and Night annoyance scores dropped 3.7 and 3.4 points on annoyance scale. (Note: retrospective Before annoyance) No statistical test reported. There is a difference in the distribution of annoyance reductions across the five airports	n.a.	Primary purpose was assessment of the overall NIP process	

**Table 13:** New/Closed Infrastructure (Type C).

Authors	Intervention & Study		*N*, Response Rate & Method	Exposure Levels		Change in Levels and Distribution of Change across Participants	Outcome Measure(s) Before and after Outcomes	Did Outcome Change with Change in Exposure? Yes/No (Significance Tested?)	Before/after Outcome Change Compared to That Estimated from an ERF	Comments	Confounders Adjusted for in Analyses
	Nature	Design		Before	After						
Brink, Wirth, Schierz, Thomann & Bauer (2008) [47]	Zurich Relocation of flights in shoulders (early morning and late evenings)	B&A study Effectively two cross sectional studies from which change sample was located 2 years gap between surveys	394 change respondents (a subgroup of the 1816 Ps who were interviewed twice 1816 Ps in first ERF study/1719 in second (Response rates 54%/36%) mail & tel	L_den_ (and others) over range 30–70 dB Calculated		Change measured in L_Aeq_ over the shoulder periods 6–9 a.m. and 9–12 p.m. Range of intervention change in this indicator was −12 dB to +12 dB, but between −3 dB and +3 dB for ~70% of the 394 Ps who experienced the intervention	ISO 0–10 numerical scale and 5 point verbal scale. HA cutoff was 8	Yes Logistic regression models of prob. High annoyance show change is a significant parameter (Effect 0.16 *p* = 0.028 for morning change; Effect = 0.16, *p* < 0.001 for evening change) in addition to L_Aeq_	ERFs for logistic regression of HA obtained from random sample around Zurich airport averaged across 2001 and 2003 surveys. Excess response ERFs developed that have L_den_ and change in L_Aeq,3 h_ as independent predictors. The ERF in this model is different to the one developed above, demonstrating an effect of change Demonstrated descriptively that Ps who experienced an increase through the intervention exhibited quite strong excess response	ERF developed in this study is approx. parallel to EU position paper ERF [14], but shifted 5–10 dB to left. Thus %HA around Zurich higher than predicted	Military aircraft noise was accounted for by exclusion; year of survey
Breugelmans et al. (2007) [48]	Amsterdam New runway opening Step-change increase in exposure Focussed on changes in outcomes	Had been 3 cross sectional surveys 1998, 2002 & 2005 2002 used as starting point for panel study Four rounds of panel survey	640 In area with forecast change >3 dB Half surveyed in different seasons 478 completed all 4 waves Mail survey	L_den_ L_night_ Calculated For the three subgroups: 53 61 55 Change in exposure is a noise indicator	57 55 54	Based on after-levels, Ps to three subgroups based on change in L_den_. >+1.5 range: 1.5 to 13.7 (mea*n* = +2.5) *n* = 118 <−1.5 range: −2.2 to −1.5 (mean = −1.9) *n* = 117 Control range: −1.4 to +1.4 (mean = 0.1) *n* = 405	ISO 0–10 scale. Reports % ‘severely’ annoyed (=%HA?)	Yes %HA does not change for control group. %HA does change for increase group (%HA changes from <40% to >60% (difference significant based on plotted Cis)	2002 panel survey before-results used to derive ERF Observed %HA control and decrease subgroups are in agreement with outcomes estimated from this ERF Observed %HA increase subgroups exceed outcome estimated from ERF Odds Ratio per 3 dB change = 0.44)—slightly less after control for confounders. Stronger association found with change over past 12 months (OR = 1.73) Excess response present for increase subgroup still present 3 years after intervention (one inconsistent result in fourth panel survey)	Part of Health Impact Assessment Schiphol Airport program Excess response was not explained by non-acoustical factors	Age, sex, ethnicity, home ownership, degree of urbanization, time of residence, living satisfaction, noise sensitivity, expectations about the airport and the neighbourhood, coping behaviour, fear for aircraft crashes, and a negative attitude towards the airport
Fidell, Silvati, and Haboly (2002) [49]	Vancouver Step change with new runway Change in aircraft operations	B/A study Independent samples, not a panel 15 mos B and 21 mos A. 3 year gap	B: 1000 A: 1067 Located in 7 areas Telephone interviews	L_dn_ 44–71 But most areas experienced 44 to 54 Calculated using INM	44–70	Ps in 5 areas experience effectively no change in exposure. One area experienced +7, another +3	Filter question as to whether bothered or annoyed in last year. If yes, then 4 point verbal annoyance scale. HA cutoff at top two points (*very, extremely*) Reported %HA each area B & A Most areas, no change. See next column	Yes +7 dB area: B: 11%; A: 52% (chi^2^ 59.8, *p* < 0.007) +3 dB area: B: 0%; A: 18% (chi^2^ 19.7, *p* < 0.007)	Author cites FICON ERF [11] For the two sites with major increase, the %HA is higher than predicted by the above ERF—and outside of a one s.d. error of the mean value of the ERF. Author notes excess response in only +7 dB area—but it is also present in the +3 dB area too Response to change in same direction as estimated by ERF, but steeper, indicating excess response	Author comments: *greater-than-predicted increase in the prevalence of annoyance cannot be attributed to change in noise exposure alone*	Attitude; dependency on airport; fear of crashes

**Table 14:** New/Closed Infrastructure (Type C).

Authors	Intervention & Study		*N*, Response Rate & Method	Exposure Levels		Change in Levels and Distribution of Change across Participants	Outcome Measure(s) before and after Outcomes	Did Outcome Change with Change in Exposure? Yes/No (Significance Tested?)	Before/after Outcome Change Compared to That Estimated from an ERF	Comments	Confounders Adjusted for in Analyses
	Nature	Design		Before	After						
Breugelmans et al. (2007) [48]	Amsterdam New runway opening Step-change increase in exposure. Focused on changes in outcomes		See study details as reported in Table 13 (annoyance) Calculated L_night_ and change in L_night_ were the exposure measures				Sleep disturbance 0–10 scale	Yes %Highly Sleep Disturbed does change for increase group	2002 panel survey before-results used to derive ERF for sleep disturbance Observed %Highly Sleep Disturbed is consistent with that estimated from above ERF Sleep disturbance response to change as estimated by ERF		Wide range of confounders incorporated into the analysis. See Table 13
Fidell, Silvati, & Haboly (2002) [49]	Vancouver Step change with new runway Change in aircraft operations		See study details as reported in Table 13 (annoyance)				Filter question, then: ‘bothered or annoyed in last year’ If Yes, Has your sleep been disturbed? Y/N Reported %sleep disturbed each area B & A Most areas, no change. See next column	Yes +7 dB area: B: 16%; A: 43% (chi^2^ 27.5, *p* < 0.007) +3 dB area: B: 5%; A: 17% (chi^2^ 8.2, *p* < 0.007)	n.a.		See Table 13

**Table 15:** New/Closed Infrastructure (Type C).

Authors	Intervention & Study		*N*, Response Rate & Method	Exposure Levels		Change in Levels and Distribution of Change across Participants	Outcome Measure(s) before and after Outcomes	Did Outcome Change with Change in Exposure? Yes/No (Significance Tested?)	Before/after Outcome Change Compared to That Estimated from an ERF	Comments	Confounders Adjusted for in Analyses
	Nature	Design		Before	After						
Hygge, Evans & Bullinger (2002) [50]	Munich Opening new airport closing old airport	Prospective cohort study +control matched om SES 3 data collection waves B (6 mos) & two A (1&2 year)	326 (mean age 10.4 years) Memory and Reading paper and pencil tests High response rates	Measured L_Aeq,24 h_ at school only Increase: 53 (*n* = 111) Decrease: 68 (*n* = 65) Control: (*n* = 107)	62 54	+9 new airport –14 old airport	Long and short term memory; reading; attention; speech perception	Yes Effects on reading, memory and speech perception, not attention. Effects disappeared when old airport closed; emerging after the new airport opened Various statistical tests including interactions.	n.a.	Children tested in soundproof caravan Suggest effects may be reversible	Confounds ruled out by design: ethnicity; mother’s education; number of family members; occupation; attrition

**Table 16:** Source Interventions (Type A).

Authors	Intervention & Study		*N*, Response Rate & Method	Exposure Levels		Change in Levels and Distribution of Change across Participants	Outcome Measure(s) before and after Outcomes	Did Outcome Change with Change in Exposure? Yes/No (Significance Tested?)	Before/after Outcome Change Compared to That Estimated from an ERF	Comments	Confounders Adjusted for in Analyses
	Nature	Design		Before	After						
Möhler et al. (1997) [51]	Germany Rail grinding to reduce railway noise emissions	B and two A. 1 mo. B; 1mo A. 3^rd^ round was 1 year after 2^nd^ round	B. 81 A. 64 A2. 46 questionnaire	L_dn_ 55–75 Calculated, with some measurement	L_dn_ 50–65	−7 to −8 dB Distribution of change across Ps not reported	0–10 total annoyance scale Reported as mean annoyance scores for group Difference in mean reported	Yes Difference between B & A = 0.6 (*t* = 2.07, df = 63, *p* < 0.05) Difference between B & A2 = 0.8 (*t* = 2.26, df = 45, *p* < 0.05) No difference between A1 & A2	n.a.		

**Table 17:** New/Closed Infrastructure (Type C).

Authors	Intervention & Study		*N*, Response Rate & Method	Exposure Levels		Change in Levels and Distribution of Change across Participants	Outcome Measure(s)	Did Outcome Change with Change in Exposure? Yes/No	Before/after Outcome Change Compared to That Estimated from an ERF	Comments	Confounders Adjusted for in Analyses
	Nature	Design		Before	After						
Lam & Au (2008) [52]	Hong Kong Opening of new 11 km urban rail line	6 mos B and two A. 2nd round 3 mo after. 3rd round was 1 year after 2nd round.	6000 invitation letters. Response rate not reported Face-to-face interviews B and A1 Telephone interview A2	Estimation of noise + validating measurements Noise mapping, validating measurement		Introduction of railway lead to very small increase in total noise exposure. L_Aeq,30 min_. 70% Ps experienced <+1 dB change. Others had +2 to +4 dB change		n.a. Results showed that original noise from road traffic overwhelmed the train noise for effectively all Ps	n.a.	There was a parallel survey over same area that experimentally manipulated information supplied to Ps about noise mitigation. This component is not reported here	

**Table 18:** Education/Communication Intervention (Type E).

Authors	Intervention & Study		*N*, Response Rate & Method	Exposure Levels		Change in Levels and Distribution of Change across Participants	Outcome Measure(s) before and after Outcomes	Did Outcome Change with Change in Exposure? Yes/No (Significance Tested?)	Before/after Outcome Change Compared to That Estimated from an ERF	Comments	Confounders Adjusted for in Analyses
	Nature	Design		Before	After						
Schreckenberg et al. (2013) [53]	Germany Rail grinding plus provision of information to Ps	BA. 3 mos B; 1–2 mos A. Part given information about rail grinding, part not given information. Randomly distributed over an information and non-information group	B: 411 A: 340 (163 informed area; 177 uninformed area) Response Rates: 73% & 83%) Repeated interviews	Not reported		Emission levels LAeq (day and night)reduced by only 1–2 dB	5 point verbal annoyance scale and range of disturbances Authors’ conclusion based on the above	Rejected that rail disturbances dropped because of noise level drop. Yes However, author suggests disturbances are less where Ps have been given information compared to not given information	n.a.		

**Table 19:** Summary of evidence from the individual studies on the effect of the intervention on health outcomes.

	Number of Papers	Evidence ^1^ That Health Outcome Changed			Observed Magnitude of Change in Health Outcome		
		YES	NO	n.a.	Magnitude *at Least* as Predicted by ERF	Excess ^2^ Response	n.a.^3^
**ROAD TRAFFIC NOISE SOURCES (33)**							
Outcome: Annoyance (23)							
A Source Intervention	9	*******		**	*******	*******	**
B Path Intervention	6	******			****	** ^?^	**
C New/Closed Infrastructure	2	**			**	**	
D Other physical	6	******					
Outcome: Sleep Disturbance (6)							
A Source Intervention	1			*			*
B Path Intervention	2	**					**
C New/Closed Infrastructure	2	**					**
D Other physical	1	*					
Outcome: Cardiovascular Effects (4)							
D Other physical	4	***	*				
**AIRCRAFT NOISE SOURCES (7)**							
Outcome: Annoyance (4)							
B Path Intervention	1	*					*
C New/Closed Infrastructure	3	***			***	***	
Outcome: Sleep Disturbance (2)							
C New/Closed Infrastructure	2	**			*		*
Outcome: Cognitive Development in Children (1)							
C New/Closed Infrastructure	1	*					*
**RAIL NOISE SOURCES (3)**							
Outcome: Annoyance (3)							
A Source Intervention	1	*					*
C New/Closed Infrastructure	1			*			*
E Education/Communication	1	*					

* Statistical significance of finding reported in the original study. * Finding interpreted by original, or current, authors based on data/tables/plots in original study. ^1^ Note that the evidence is indirect for Interventions Type D (Other Physical). ^2^ Excess response occurs where the total difference between the observed before and after outcomes is greater than the magnitude of the change in response estimated from an ERF, for a given change in exposure. ^3^ n.a. = not applicable/not available: no change in exposure or not reported. ^?^ = unclear finding.

**Table 20:** Model protocol for intervention studies. After Brown (2015) [17].

Sequential Measurements	Before_−1_	Before_0_	After_1_	After_2_ ...
Time	t_−1_	t_0_	t_1_	t_2 …_
noise exposure	L_−1_	L_0_	L_1_	L_2 …_
**Effect Measures (or Respondent Attribute Measures)**				
annoyance	A_−1_	A_0_	A_1_	A_2_
activity interference	Act_1_	Act_0_	Act_1_	Act_2_
retrospective annoyance			RA_01_^1^	RA_02_
noise sensitivity	Sens_-1_	Sens_0_	Sens_1_	Sens_2_
attitudes to authorities etc.	Ats_−1_	Ats_0_	Ats_1_	Ats_2_
opinion of neighbourhood	Neigh_−1_	Neigh_0_	Neigh_1_	Neigh_2_
coping strategies	Cop_−1_	Cop_0_	Cop_1_	Cop_2_
prior knowledge	…	X_10_^2^	…	…
expectations	…	Y_10_^2^	…	…
**Steady-state Controls**	Before Control		After Control	

^1^ RA_01_ is a respondent’s retrospective assessment of annoyance at t_1_ of conditions that existed at t_0_. ^2^ X_10_ and Y_10_ are respondent’s prior knowledge, and expectations, at t_0_, of conditions that will exist at t_1_. Other non-acoustic factors may have to be added.

## Supplementary Materials

The following are available online at www.mdpi.com/1660-4601/14/8/873/s1, Table S1: Previous narrative review papers on transport noise source interventions and effects, Table S2: Key search terms (in title, abstract and/or keywords), Table S3: Studies excluded based on full-text reading, Tables S4 and S5: Modelled outcomes of hypothetical interventions, Tables S6–S15: GRADE Tables for quality of evidence for various combinations of source, intervention type and outcome, and of individual studies, Tables S16–S29: Assessment of the risk of bias in the individual studies, Tables S30–S39: Hospital Noise and PLD/Music Venues/Other Sources Interventions.
