# Supplementary material for: WHO Environmental Noise Guidelines for the European Region: A Systematic Review of Transport Noise Interventions and Their Impacts on Health
Source: Int J Environ Res Public Health. 2017 Aug 3;14(8):873. doi: 10.3390/ijerph14080873 (PMC5580577; doi:10.3390/ijerph14080873)
Supplement: Supplementary file 1 [file ijerph-14-00873-s001.pdf]

International Journal of
Environmental Research and Public Health

MDPI

Supplementary Materials: WHO Environmental Noise Guidelines for the European Region: A Systematic Review of Transport Noise Interventions and Their Impacts on Health

Supplementary File 1

Table S1. Previous narrative review papers on transport noise source interventions and effects on (primarily) annoyance and sleep disturbance.

Review Paper	Annotations
Köhler, J., Ruijsbroek, A. & van Poll, R. (2009) [9]	Review of the (perceived) influence (annoyance and sleep disturbance) and effectiveness of noise mitigating measures for dwellings aimed at reducing road and air traffic noises. Literature on this topic within the timeframe of 1980–2005 noted to be scarce. Six field and four semi-experimental studies were identified. Seven of the ten studies showed positive results due to measures taken, though the small number of cases did not allow for drawing firm conclusions. Three of the ten reviewed studies, among them the largest observational study comprising nearly 1000 respondents, concluded that insulation has no, or only a moderate effect on reducing annoyance/ satisfaction or on improving the sleep quality. From the review it appeared that several (non-acoustical) factors influenced the effectiveness of the insulation.<br>Half of the studies reported were for insulation against road traffic noise. Most studies have a small sample size and all except one study addressed the effectiveness of insulation on sleep quality. The gain in terms of a reduction of exposure levels varied between −8 and −34 dB (A) after the intervention. The studies all indicated some modest improvement of the sleep quality, and/or the reduction of number of bed movements (motility) during sleep in three of the four studies. In the largest studies (N = 381) only satisfaction with the insulation and acceptance of it was measured.<br>The other half of the studies concerned aircraft noise, but most were non-peer-reviewed ministerial reports. One study compared the prevalence of annoyance in insulated versus non insulated homes. Sample size was 936. People were asked about their annoyance only after the intervention. No information was provided about the gain in dB after insulation.
Brown A.L. & van Kamp I. (2009) [15]<br><br>Brown A.L. & van Kamp I. (2009) [54]	These papers describe the 2009 Brown and van Kamp review of the literature on annoyance responses to step changes in noise exposure from transport sources. This included some forty studies that reported investigations of step changes in level. The weight of evidence was that, for road traffic, there is a change effect in addition to an exposure effect. The change effect is manifest as an excess response to the new noise exposure over that predicted from steady-state exposure–response curves. Excess response was found for changes in road traffic noise—in noise annoyance responses though not in activity interference responses. This was only for change in exposure resulting from an increment or decrement in source levels (Type 1 changes) rather than from the insertion of barriers or other path mitigation interventions (Type 2 changes). The magnitude of the excess response found in Type 1 road traffic noise interventions covered a decibel-equivalent range from greater than −20 dB following a decrease in exposure to +15dB following an increase in exposure. The trend in the data was that the magnitude of the change effect had the decibel-equivalent of the magnitude of the change in exposure itself, and in the same direction. There were insufficient or limited studies of aircraft noise changes in annoyance to make any observation of a change effect for aircraft noise. The companion paper catalogues and reviews the different explanations for excess reaction to change in noise.<br>Several of the individual studies in this review fulfilled the criteria for this review of interventions and have been included below.
Laszlo, H. E., McRobie, E. S., Stansfeld, S. A. & Hansell, A. L. (2012) [57]	This review updates the previously reported review of change studies by Brown and van Kamp (2009a, 2009b) including factors affecting annoyance in both changed and steady-state conditions. The review also documents changes in other outcomes to changed noise conditions. However, it duplicates most studies included in previous reviews and the review does not provide additional insights into the nature and magnitude of change effects or potential mechanism of change effects.
van Kamp, I. & Brown, A.L. (2013) [58]	This was a partial update of the Brown & van Kamp (2009a, 2009b) reviews considering more recent change studies. The focus is on further evidence for the existence of the change effect and its explanations. The intervention and change studies conducted since the original reviews generally confirm, certainly do not conflict with, the above observations - though the number of new studies quantifying the change effect was insufficient to add to the previously quantitative estimates of the magnitude of excess response by Brown & van Kamp (2009a).

Supplementary File 2

Table S2. Key search terms (in title, abstract and/or keywords).

Exposure	1. noise*or ((noise sensitivity or noise perception) or noise/) and (hearing or sound*).<br>2. (traffic or transport* or road or roads or road-traffic or road-transport or automobile* or vehicle* or vehicular movements or motorcycle* or tram or train or trains or railway* or railroad* or airplane* or aeroplane* or aircraft* or airport* or air-traffic or nightflights or night flights).<br>3. exp transportation/ or exp motor vehicles/ or exp railroads/ or exp aviation/ or environmental exposure/ or environmental health/ or environment<br>4. (environment or environmental or windfarm* or wind farm* or windmill* or wind turbine* or wind park* or wind turbine* or turbine noise*).<br>5. (music or electronic devices* or listening devices or headphone* or festival* or disco* or recreation* or leisure) or recreation/ or leisure activities/<br>6. hearing loss, noise induced/<br>7. 1 and (2 or 3 or 4 or 5 or 6)<br>8. (noise pollution or noise exposure).ti. or transportation noise
Health effects	9. adverse effects. (annoyance or disturbance or nuisance or bother*).<br>10. (health or mortality or morbidity or wellbeing) or health/ or health status/ or mental health/ or quality of life/ or public health/<br>11. (stress or asthma or respiratory or blood pressure or heart rate* or cardiovascular).tw. or stress, psychological/ or stress, physiological/ or emotions/ or asthma/ or child behavior/ or blood pressure/ or heart rate/<br>12. (cognitive performance or cognitive impairment or cognition or cognitive development or cognitive effects or memory or recognition or loudness perception or reading or pre-reading or school performance or performance or comprehension or annoyance or (disturbance adj3 daily activity*) or emotion* or stress or perception or speech or intelligibility or hearing impairment or hearing loss or tinnitus)<br>13. cognition/ or cognition disorders/ or memory/ or reading/ or mental recall/ or recognition, psychology/ or loudness perception/ or perception/ or auditory perception/ or comprehension/ or adaptation, psychological/ or speech intelligibility/ or hearing disorders/ or hearing loss/ or tinnitus/<br>14. (sleep or insomnia or awakening*)or exp sleep/ or exp sleep disorders/ or sleep deprivation/ or wakefulness/<br>15. (reproductive outcome* or pregnancy outcome* or birth outcome* or birth weight) or pregnancy outcome/ or birth weight/<br>16. (7 or 8) and (9 or 10 or 11 or 12 or 13 or 14 or 15)
Intervention	17. (prevention or preventive or prevent or preventative or preventing or intervening or intervention* or mitigation or measures or reduction or reducing or reduce or improving or minimizing or program* or campaign* or project* or policy or policies or strategy* or guidelines or directive* or community response or public health response)
Design	No restrictions
Time period	1980–2014
Language	No restrictions

Supplementary File 3

Table S3. Studies Excluded Based on Full-text Reading.

**ROAD TRAFFIC NOISE: STUDIES REPORTING A CHANGE IN HEALTH OUTCOMES**	
Dravitzki, V. K., & Wood, C. W. B. (2003) [59]<br>Walton, D., & Dravitzki, V. (2003) [60]	Change in annoyance from a road surface intervention<br>This is a study into the effect of a road surface change on noise level, annoyance and behaviour in adjacent residents. The study included a before/ after measurement of 138 respondents at 12 sites and a follow up after 6 months. The paper does not meet the criteria because interventions reported only a change in drive-by levels of vehicles ranging from -7 to +6dBA changes. L_{Aeq24} levels are reported only partially. Annoyance was measured using the ISO standard 10 point scale, and the general community reaction scale of Job et al, 2001. Results show a considerable decrease in annoyance with 7dB reduction and increases even with increase in noise of 1 dB.

Eberhardt, J.L. & Akselson, K.R. (1987) [61]	Experimental intervention study on insulation effects, excluded because the intervention was only temporary
Griefahn, B., Marks, A., & Robens, S. (2008) [62]	This experimental study into the effect of curfews on sleep disturbance was excluded because it was a laboratory study. The noise intervention studied included time frame curfews. It was shown that curfews were only effective at the end of the subjective sleep period. Also it was demonstrated that even short periods of noise had adverse effects on sleep at the end of night.
Klæboe, R., Kolbenstvedt, M., Fyhri, A. & Solberg, S. (2005) [63]	This paper investigates whether an adverse neighbourhood soundscape—noisy areas along roads in the immediate neighbourhood of the dwelling—contributes to residential noise annoyance. However, the evidence for the existence of such a soundscape effect on annoyance is based on annoyance *outside the dwelling*. While indoor annoyance is also measured in the study, this does not appear to have been presented in terms of how it is influenced by neighbourhood soundscape.
Gomez-Jacinto, L. & Moral-Toranzo, F. (1999) [64]	Insulation study. Excluded because inadequate reporting of outcomes.
Harupa, A., & Richard, J. (2000) [65]	This study describes the noise abatement planning in a German city. Some noise reducing measures were evaluated in a post-measuerment only, therefore not included in the review.
Klæboe, R., Kolbenstvedt, M., Lercher, P. and Solberg, S. (1998) [66]	Large area-wide traffic reductions in Oslo as a result of tunnel construction. Presumably also large traffic noise reductions. Eight sub-areas. Large surveys (n=898, 564 and 588) in 1987, 1994 and 1996 respectively. Reported exposure–response function for each survey. Large excess response. Calculated noise levels. Drop in percentage highly annoyed at 60 dB from 30% to 12% over the three survey. While noise levels had been calculated at 157 locations, study excluded because it did not report any information on the reductions in exposure. Also excluded because noise outcome (highly annoyed) was assessed *outside* people's apartments. Paper's focus was primarily on explaining observed excess response (hypotheiseized as an area effect.
Mital, A., & Ramakrishnan, A. S. (1997) [67]	This study addressed the effect of noise barrier installation on annoyance. The study does not meet the inclusion criteria because of inadequate reporting of noise levels measures, measures of human response, and the sampling method used.
Öhrström E. & Björkman, M. (1983) [68]	This is a small-size ecological intervention study on the effect of insulation on sleep disturbance. Study too small (sample size: 3) to draw any firm conclusions
Tulen, J.H.M., Kumar, A. & Jurriëns, A.A. (1986) [69]	Experimental intervention study on insulation effects, excluded because the intervention was only temporary
Utley, W. A., Buller, I. B., Keighley, E. C., & Sargent, J. W. (1986) [70]	This study measured the effectiveness and satisfaction with level reduction from insulation of the homes. The study does not meet the criteria since it is primarily concerned with resident satisfaction with the package of noise insulation. Dissatisfaction with traffic noise was reported, but not in a useable way.
Wilkinson, R.T. & Campbell, K.B. (1984) [71]	Experimental intervention study on insulation effects, excluded because the intervention was only temporary
**AIRCRAFT NOISE: STUDIES REPORTING A CHANGE IN HEALTH OUTCOMES**	
Cohen, S., et al. (1981) [72]	This study (see also Cohen et al. 1980 and 1981b) was not included in the review since it is not an intervention study and does it meet the criteria set in the protocol fully. It does draw conclusions about the clinical or policy significance of the data from a cross-sectional and longitudinal study and the effectiveness of short term noise insulation
Fidell, S., Horonjeff, R., Teffeteller, S., & Pearsons, K. (1981) [73]	This paper addresses the effect of a temporary change in flight paths. Therefor it is not included
Fidell, S., Pearsons, K., Tabachnick, B. G., & Howe, R. (2000) [74]	This study investigated the effect of changes at three airports (One airport closing, another opening, and thirdly including temporary changes during the Olympic Games. Detected changes in noise events (automatic detection) both before and after change and inside and outside dwellings. The study was excluded on the basis of poor specification of the intervention re events from overflights.
Klæboe, R. (2005) [75]<br>Krog, N. H., & Engdahl, B. (2004) [76]<br>Krog, N. H., & Engdahl, B. (2005) [77]	These three papers pertain to changes in noise in recreational areas—a setting which is not included in our WHO brief.
Seabi, J. (2013) [78]	This study investigated the effects of relocation of an airport on annoyance and health reactions in children. Analysis is not based on a change of levels, but is based on difference of effects in high and low exposed children.
Stansfeld, S., Hygge, S., Clark, C., & Alfred, T. (2010) [79]	This study partly included the effects of relocation of an airport on childrens cognition (Munich Arport). The rest of the paper pertains to the RANCH study, which was not an intervention study. Paper does not add to the paper of Hygge et al. (2002) [50].

Van Kamp, I., Houthuijs, D., Van Wiechen, C., & Breugelmans, O. (2007) [80]	This paper and a paper by same authors in 2006 provide no evidence of any effect associated with the change/intervention on mental health or cardiovascular disease even though there was an intervention (extension of the airport).
Wirth et al. (2006) [81]	This conference paper duplicates the Brink et al (2008) paper [47] which is included.
**RAILWAY NOISE: STUDIES REPORTING A CHANGE IN HEALTH OUTCOMES**	
Bronzaft A.L. (1981) [82]	Study excluded based on limited reporting of change in noise exposures as a result of rail source level reduction and absorptive ceilings in classrooms.
Kawabata, T. (1991) [83]	This paper addresses a change in source levels of the Shinkansen by increasing its speed and its cognitive effects on schoolchildren: The paper is only available in Japanese.
Oka, S., Tetsuya, H., Yano, T., & Murakami, Y. (2012) [84]	In this study the community response was measured in terms of annoyance (noise and vibration) after the opening of the Kyushu Shinkansen line that ran largely parallel to a conventional rail line. Noise and vibration exposures were slightly decreased after the opening due to lower levels from the high speed Shinkansen than from the conventional trains. Results showed a decrease of percentage highly annoyed after the opening of the Kyushu Shinkansen line. However it is not possible to readily describe the nature and extent of the intervention and therefore excluded.
Ohrstrom, E. (1997) [85]	Excluded as study only examines vibration.
**OTHER (MULTIPLE TRANSPORT SOURCE, AND MILITARY WEAPON SOURCES) NOISE STUDIES REPORTING A CHANGE IN HEALTH OUTCOMES**	
Nykaza, E. T., Pater, L. L., Melton, R. H., & Luz, G. A. (2009) [86]	This paper addresses the association between blast noise (military firing) and sleep. This source is not included in the WHO protocol.
Steensberg, J. (1999) [87]	This is a general paper on 'history' of Danish noise policy and therefore it was excluded.
**STUDIES REPORTING A CHANGE IN NOISE LEVELS FROM ROADWAYS/RAILWAYS/AIRPORT S**	
The papers below were all excluded as they only indicate change in noise levels and not change in exposure or a change in effect.	
Berge, T., & Storeheier, S. Å. (2009) [88]	Level change due to low noise pavements but no exposure level change nor effects.
Brown, A. L., Tomerini, D., Carroll, J., & Scott, N. D. (2009) [89]	Longitudinal level change response to reduced trucks but no exposure level change nor effects.
Kim, S. K., Park, W. J., & Lee, K. H. (2014) [90]	Road traffic noise reduction by pavements but no exposure level change nor effects.
Khardi, S., Abdallah, L., Konovalova, O., & Houacine, M. (2010) [91] (See also: Khardi, Abdallah(2012) [92])	Modelling flight paths to reduce exposure but no exposure level change nor effects.
Lakušić, S., & Ahac, M. (2012) [93]	Noise reduction measures for railway but no exposure level change nor effects.
Qing-fei et al. (2007) [94]	Effect of different plant communities on roadside levels
van Renterghem, T., Attenborough, K., Maennel, M., Defrance, J., Horoshenkov, K., Kang, J., . . . Yang, H. S. (2014) [95]	Road traffic noise reduction by hedges but no exposure level change nor effects.
**INTERVENTION STUDIES REPORTING CHANGE IN ATTITUDES/KNOWLEDGE/INTENTIONS**	
Maris, E., Stallen, P. J., Vermunt, R., & Steensma, H. (2007) [96]	This is a laboratory experiment into a change in annoyance by manipulating 'fairness' of sound management.
**INTERVENTIONS STUDIES REPORTING MODELLED CHANGE IN EXPOSURE OR EFFECT**	
Lee, P. J., Kim, Y. H., Jeon, J. Y., & Song, K. D. (2007) [97]	This study models only the noise levels and not the exposure levels and therefore excluded.
Avsar, Y., & Gumus, B. D. (2011) [98]	This study models only the noise levels and not the exposure levels and therefore excluded.
Dintrans, A., & Préndez, M. (2013) [99]	This study models future annoyance and sleep disturbance on road network with volume speed and road surface scenarios but does not give information about a change in exposure nor in effects. Therefore excluded.
Giering, K., & Augustin, S. (2011) [100] Giering, K., Augustin, S., & Strünke-Banz, S. (2013) [101]	This study calculates a RailwayNoiseIndex RNI for annoyance, and one for additional awakenings. There seems to be a link to disposition of dwellings near rail, topography and barriers? The index does not provide the infromation needed for comparsion and evaluation: therefore excluded.

Supplementary File 4

Modelled Outcomes of Hypothetical Interventions

1. Modelled Outcomes

Three of the individual studies identified through the search, two for road traffic and one for aircraft noise, modelled the outcomes from hypothetical interventions. While studies of this type do not provide *evidence* of the effect of interventions, and hence are not included in the body of this report, they effectively provide important information of the likely extent and magnitude of change in outcomes. Such modelling constitutes a sensitivity analyses to potential interventions, which can assist in the allocation of resources for interventions, and also could assist in the design of future intervention studies. Scenario analysis may be of particular relevance to local authorities and to other implementation agencies. The results of modelling of hypothetical interventions in these three studies are reported in this section. It should be noted that the authors did not conduct a comprehensive search for such studies.

2. Road Traffic Noise: Modelled Changes in Exposure/Effect

While there were no individual studies that *reported change in the noise exposure of a specific population of interest*, two studies modelled the effect of hypothetical interventions, reporting either modelled change in exposure of populations of interest or modelled change in their health outcomes.

Summary: Information from modelled road traffic noise interventions, Table S4.

The two available studies modelled exposure of urban populations, and one modelled the percentage of the urban population that was highly annoyed - based on this exposure estimate. The modelling involved a combination of hypothetical interventions. One study focussed on interventions of traffic speed and/or traffic volume reductions. Results of the interventions were reported as the percentage of change (increase and decrease) at population levels, within the 5 dB exposure bands, for each intervention. Reductions in the percentages of the population exposed to L_{den} greater than 70dB ranged from −2% to −7.2% depending on the hypothetical intervention tested.

The second study examined interventions involving hypothetical combinations of quiet tires, roads and quiet cars for three EU cities. Results of the interventions were reported as the percentage of decrease at population levels, for the percentages of the population highly annoyed. Reductions in the percentages highly annoyed ranged from -1% to -7% depending on the hypothetical intervention tested. Combined interventions were shown to be more effective than any single intervention. The study took porous road surfaces into account.

3. Aircraft Noise: Modelled Change in Exposure/Effect

3.1. Included Paper

Summary: Information from modelled aircraft noise intervention, Table S5 below.

For the relatively small airport at Pisa, the study modelled the aircraft noise exposure of the urban populations. It also modelled the number of people who would be highly annoyed, or highly sleep disturbed, under five different hypothetical mitigation strategies regarding aircraft operations. It is not the particular strategies at this airport, or the estimates generated, that are of interest in this review, but a demonstration of the ability to estimate likely consequences, in health outcome terms, of a variety of environmental noise interventions.

Table S4. SOURCE 'INTERVENTIONS' (Type A).

**Authors**	**Intervention & Study**		***N*, Response Rate & Method**	**Exposure Levels**	**Change in Levels and Distribution of Change**	**Outcome Measure**	**Comments**
	Nature	Design					
Murphy & King (2011) [102]	Dublin Scenario analysis modelling population exposures from different road traffic noise mitigation strategies	Mitigation strategies:<br>traffic flow reduction;<br>speed reduction;	Population level estimates	L_{den}, L_{night}<br>Modelled.<br>Some measurement validation	Estimated proportion of population exposed<br>Before:<br>L_{den} > 70 27% L_{night} > 40 85%<br>Reductions in population %ages achieved by:<br>L_{den} L_{night}<br>10% travel reduction −1.0% −2.9%<br>20% travel reduction −4.9% −7.2%<br>10% speed reduction −0.2% −2.0%<br>20% speed reduction −4.8% −3.7%<br>Greater changes in reduction of high exposures	n.a.	Conversion used:<br>L_{den} = 0.86 x $L_{A10,18h}$ +9.86
Roovers & Van Blokland (2005) [103]	Three European cities:<br>Modelling scenarios of reducing road surface noise	Mitigation strategies for combined silent tyres, silent vehicles and silent road surfaces	Population level estimates.<br>Modelled three policy scenarios:<br>1. no effort<br>2. extra effort<br>3. much extra effort	Modelled L_{den} at all dwellings in urban area in 1 dB classes using 2005 as the base year.	Modelled levels in each city for Scenarios 1 to 3 for year 2010.<br>After levels not shown – results reported at percentages of urban population HA and Annoyed	Modelled %HA & %A for free-flow situations using ERF (from *EU's Future Noise Policy*, WG2: 20 Feb, 2002) + another developed for interrupted flow conditions.<br>%HA of urban population reduced by:<br>Scenario 1: 0.7% to 1.9%<br>Scenario 2: 2.2% to 5.1%<br>Scenario 3: 3.0% to 6.7%	GIS approach needs to be validated.

Table S5. SOURCE INTERVENTIONS (Type A).

Authors	Intervention & Study		N, Response Rate & Method	Exposure Levels	Change in Levels and Distribution of Change	Outcome Measure(s)	Comments
	Nature	Design					
Licitra, Gagliardi, Fredianelli & Simonetti (2014) [104]	Pisa<br>Scenario analysis modeling population exposures from different aircraft noise mitigation strategies	Mitigation strategies:<br>Change in aircraft procedures;<br>Change in departure profile;<br>Combined strategies	Population level estimates	INM modelling of L_{den} & L_{night} across urban population	Estimated numbers of people exposed to L_{den} in 5 dB bands calculated for 2011 and 2013 scenarios	Models numbers of HA and Highly Sleep Disturbed in urban population for five different mitigation scenarios.	Needs validation

Supplementary File 5

GRADE Tables for Quality of Evidence for Various Combinations of Source, Intervention Type and Outcome

Table S6. GRADE Table for the quality of evidence for road traffic noise interventions Type A, B, & C (Source, Path and New/Closed Infrastructure Interventions) and annoyance.

Domains	Criterion	Assessment	Downgrading
Start Level	Model protocol for longitudinal study design for noise interventions is reported in Section 9.2.2 of systematic review = high	Most are before-and-after (uncontrolled) longitudinal studies, some were controlled before-and-after studies, one an interrupted time series study.	High quality
1. Study Limitations	Majority of studies carry risk of bias	Risk of bias inherent in most intervention studies as change in exposure due to intervention usually varies across participants, and participant selection rarely possible.	Downgrade one level
2.Inconsistency	Conflicting results; high I^2	Highly consistent finding that intervention resulted in a change in the health outcome (annoyance), and in the expected direction.; I^2 not possible to determine	No reason for downgrading
3. Directness	Direct comparison; same PECO	The studies assess the impact of interventions along the pathway between environmental noise and human health.	No reason for downgrading
4. Precision	Confidence interval contains 25% harm or benefit	Meta-analysis not possible. Individual studies consistently report numerical results with statistical significance.	No reason for downgrading
5. Publicatn. Bias	Funnel plot	Unclear, not possible to determine.	No downgrade
Overall judgment			Moderate quality
6. Dose–response	Significant trend	Observed magnitude of change in health outcome as predicted by relevant ERF, or demonstrates a significant excess response.	Upgrade one level
7. Magnitude of effect	RR > 2	Not possible to assess.	No reason for upgrade
8. Confounding adjusted	Effect in spite of confounding working towards the nil	Not possible to assess	No reason for upgrade
Overall Judgment			High quality

Table S7. GRADE Table for the quality of evidence for road traffic noise interventions Type D (Other Physical Dimension Interventions) and annoyance.

Domains	Criterion	Assessment	Downgrading
Start Level	Model protocol for longitudinal study design for noise interventions is reported in Section 9.2.2 of systematic review of interventions = high	Most are cross sectional studies.	Low quality
1. Study Limitations	Majority of studies carry risk of bias	Risk of bias	Downgrade one level
2. Inconsistency	Conflicting results; high I^2	Consistent finding that intervention resulted in a change in the health outcome (annoyance), and in the expected direction.; I^2 not possible to determine	No reason for downgrading
3. Directness	Direct comparison; same PECO	The studies indirectly assess the impact of the interventions along the pathway between environmental noise and human health.	No reason for downgrading
4. Precision	Confidence interval contains 25% harm or benefit	Meta-analysis not possible.	No reason for downgrading
5. Publication Bias	Funnel plot indicates	Unclear, not possible to determine	No downgrade
Overall judgment			Very low quality
6. Dose–response	Significant trend	Not possible to assess.	No reason for upgrade
7. Magnitude of effect	RR > 2	Not possible to assess.	No reason for upgrade
8. Confounding adjusted	Effect in spite of confounding working towards the nil	Not possible to assess	No reason for upgrade
Overall Judgment			Very low quality

Table S8. GRADE Table for the quality of evidence for road traffic noise interventions Type B & C (Path and New/Closed Infrastructure Interventions) and sleep disturbance.

Domains	Criterion	Assessment	Downgrading
Start Level	Model protocol for longitudinal study design for noise interventions is reported in Section 9.2.2 of systematic review of interventions = high	Before-and-after (uncontrolled) longitudinal studies, or controlled before-and-after studies,	High quality
1. Study Limitations	Majority of studies low quality	All studies carry risk of bias	Downgrade one level
2. Inconsistency	Conflicting results; high I^2	Consistent finding that intervention resulted in a change in sleep disturbance, and in the expected direction.; I2 not possible to determine	No reason for downgrading
3. Directness	Direct comparison; same PECCO	The studies assess the impact of interventions along the pathway between environmental noise and human health.	No reason for downgrading
4. Precision	Confidence interval contains 25% harm or benefit	Meta-analysis not possible. Individual studies consistently report numerical results with statistical significance.	No reason for downgrading
5. Publication Bias	Funnel plot indicates	Unclear, not possible to determine.	No downgrade
Overall judgment			Moderate quality
6. Dose–response	Significant trend	Not possible to assess	No reason for upgrade
7. Magnitude of effect	RR > 2	Not possible to assess	No reason for upgrade
8. Confounding adjusted	Effect in spite of confounding working towards the nil	Not possible to assess	No reason for upgrade
Overall Judgment			Moderate quality

Table S9. GRADE Table for the quality of evidence for road traffic noise interventions Type D (Other Physical Dimension Interventions) and sleep disturbance.

Domains	Criterion	Assessment	Downgrading
Start Level	Model protocol for longitudinal study design for noise interventions is reported in Section 9.2.2 of systematic review of interventions = high	Cross sectional study.	Low quality
1. Study Limitations	Majority of studies carry risk of bias	Risk of bias	Downgrade one level
2. Inconsistency	Conflicting results; high I^2	Consistent finding that intervention resulted in a change in the health outcome, and in the expected direction.; I^2 not possible to determine	Downgrade one level as single study
3. Directness	Direct comparison; same PECO	The studies indirectly assess the impact of the interventions along the pathway between environmental noise and human health.	No reason for downgrading
4. Precision	Confidence interval contains 25% harm or benefit	Meta-analysis not possible.	No reason for downgrading
5. Publication Bias	Funnel plot indicates	Unclear, not possible to determine.	No downgrade
Overall judgment			Very low quality
6. Dose–response	Significant trend	Not possible to assess.	No reason for upgrade
7. Magnitude of effect	RR > 2	Not possible to assess.	No reason for upgrade
8. Confounding adjusted	Effect in spite of confounding working towards the nil	Not possible to assess	No reason for upgrade
Overall Judgment			Very low quality

Table S10. GRADE Table for the quality of evidence for road traffic noise interventions Type D (Other Physical Dimension Interventions) and cardiovascular effects.

Domains	Criterion	Assessment	Downgrading
Start Level	Model protocol for longitudinal study design for noise interventions is reported in Section 9.2.2 of systematic review of interventions = high	Cross-sectional studies	Low quality
1. Study Limitations	Majority of studies low quality	All studies carry risk of bias	Downgrade one level
2. Inconsistency	Conflicting results; high I^2	Consistent finding that intervention resulted in a change in the health outcome, and in the expected direction.; I2 not possible to determine	No downgrade
3. Directness	Direct comparison; same PECCO	The studies indirectly assess the impact of the interventions along the pathway between environmental noise and human health.	No reason for downgrading
4. Precision	Confidence interval contains 25% harm or benefit	Meta-analysis not possible. Individual studies consistently report numerical results with statistical significance.	No reason for downgrading
5. Publication Bias	Funnel plot indicates	Unclear, not possible to determine.	No downgrade
Overall judgment			Very low quality
6. Dose–response	Significant trend	Not possible to assess	No reason for upgrade
7. Magnitude of effect	RR > 2	Not possible to assess	No reason for upgrade
8. Confounding adjusted	Effect in spite of confounding working towards the nil	Not possible to assess	No reason for upgrade
Overall Judgment			Very low quality

Table S11. GRADE Table for the quality of evidence for aircraft noise interventions Type B (Path Interventions) and annoyance.

Domains	Criterion	Assessment	Downgrading
Start Level	Model protocol for longitudinal study design for noise interventions is reported in Section 9.2.2 of systematic review of interventions = high	Before and after but with retrospective assessment.	Very low quality
1. Study Limitations	Majority of studies low quality	All studies carry risk of bias	Downgrade one level
2. Inconsistency	Conflicting results; high I^2	I^2 not possible to determine	Downgrade one level as single study
3. Directness	Direct comparison; same PECCO	The studies assess the impact of the interventions along the pathway between environmental noise and human health.	No reason for downgrading
4. Precision	Confidence interval contains 25% harm or benefit	Few studies report numerical results and CIs.	Downgrade one level
5. Publication Bias	Funnel plot indicates	Unclear, not possible to determine.	No downgrade
Overall judgment			Very low quality
6. Dose–response	Significant trend	Not possible to assess	No reason for upgrade
7. Magnitude of effect	RR > 2	Not possible to assess	No reason for upgrade
8. Confounding adjusted	Effect in spite of confounding working towards the nil	Not possible to assess	No reason for upgrade
Overall Judgment			Very low quality

Table S12. GRADE Table for the quality of evidence for aircraft noise interventions Type C (New/Closed Infrastructure Interventions) and annoyance.

Domains	Criterion	Assessment	Downgrading
Start Level	Model protocol for longitudinal study design for noise interventions is reported in Section 9.2.2 of systematic review of interventions = high	One study is a controlled before-and-after longitudinal study; another an interrupted time series study, but a third is based on cross-sectional surveys.	Moderate quality
1. Study Limitations	Majority of studies low quality	Risk of bias inherent in most intervention studies as change in exposure due to intervention usually varies across participants, and participant selection rarely possible.	Downgrade one level
2. Inconsistency	Conflicting results; high I^2	Highly consistent finding that intervention resulted in a change in the health outcome (annoyance), and in the expected direction.; I^2 not possible to determine	No reason for downgrading
3. Directness	Direct comparison; same PECCO	The studies assess the impact of interventions along the pathway between environmental noise and human health.	No reason for downgrading
4. Precision	Confidence interval contains 25% harm or benefit	Meta-analysis not possible. Individual studies consistently report numerical results with statistical significance.	No reason for downgrading
5. Publication Bias	Funnel plot indicates	Unclear, not possible to determine.	No downgrade
Overall judgment			Low quality
6. Dose–response	Significant trend	Observed magnitude of change in health outcome as predicted by relevant ERF, or in many cases demonstrates a significant excess response.	Upgrade one level
7. Magnitude of effect	RR > 2	Not possible to assess.	No reason for upgrade
8. Confounding adjusted	Effect in spite of confounding working towards the nil	Not possible to assess	No reason for upgrade
Overall Judgment		.	Moderate quality

Table S13. GRADE Table for the quality of evidence for aircraft noise interventions Type C (New/Closed Infrastructure Interventions) and sleep disturbance.

Domains	Criterion	Assessment	Downgrading
Start Level	Model protocol for longitudinal study design for noise interventions is reported in Section 9.2.2 of systematic review of interventions = high	One study is an interrupted time series study, the other is based on cross-sectional surveys.	Moderate quality
1. Study Limitations	Majority of studies low quality	Risk of bias inherent in most intervention studies as change in exposure due to intervention usually varies across participants, and participant selection rarely possible.	Downgrade one level
2. Inconsistency	Conflicting results; high I^2	I^2 not possible to determine	No reason for downgrading
3. Directness	Direct comparison; same PECCO	The studies assess the impact of interventions along the pathway between environmental noise and human health.	No reason for downgrading
4. Precision	Confidence interval contains 25% harm or benefit	Meta-analysis not possible. Some Individual studies report numerical results with statistical significance.	No reason for downgrading
5. Publication Bias	Funnel plot indicates	Unclear, not possible to determine.	No downgrade
Overall judgment			Low quality
6. Dose–response	Significant trend	Not possible to assess	No reason for upgrade
7. Magnitude of effect	RR > 2	Not possible to assess	No reason for upgrade
8. Confounding adjusted	Effect in spite of confounding working towards the nil	Not possible to assess	No reason for upgrade
Overall Judgment			Low quality

Table S14. GRADE Table for the quality of evidence for aircraft noise interventions Type C (New/Closed Infrastructure Interventions) and cognitive development of children.

Domains	Criterion	Assessment	Downgrading
Start Level	Model protocol for longitudinal study design for noise interventions is reported in Section 9.2.2 of systematic review of interventions = high	Before and after longitudinal study using prospective cohort with controls	High quality
1. Study Limitations	Risk of bias	Low risk of bias	No downgrade
2. Inconsistency	Conflicting results; high I^2	I^2 not possible to determine	Downgrade one level
3. Directness	Direct comparison; same PECCO	The studies assess the impact of interventions along the pathway between environmental noise and human health.	No reason for downgrading
4. Precision	Confidence interval contains 25% harm or benefit	Meta analysis not possible. Numerical result reported.	No downgrade
5. Publication Bias	Funnel plot indicates	Unclear, not possible to determine.	No downgrade
Overall judgment			Moderate quality
6. Dose–response	Significant trend	Not possible to assess	No reason for upgrade
7. Magnitude of effect	RR > 2	Not possible to assess	No reason for upgrade
8. Confounding adjusted	Effect in spite of confounding working towards the nil	Not possible to assess	No reason for upgrade
Overall Judgment			Moderate quality

Table S15. GRADE Table for the quality of evidence for railway noise interventions Type A, C, & E (Source, New/Closed Infrastructure, and Education Interventions) and annoyance.

Domains	Criterion	Assessment	Downgrading
Start Level	Model protocol for longitudinal study design for noise interventions is reported in Section 9.2.2 of systematic review of interventions = high	Most are cross sectional studies.	Low quality
1. Study Limitations	Majority of studies carry risk of bias	Risk of bias	Downgrade one level
2. Inconsistency	Conflicting results; high I^2	I^2 not possible to determine	Downgrade one level
3. Directness	Direct comparison; same PECCO	The studies assess the impact of interventions along the pathway between environmental noise and human health.	No reason for downgrading
4. Precision	Confidence interval contains 25% harm or benefit	Some numerical results.	Downgrade one level
5. Publication Bias	Funnel plot indicates	Unclear, not possible to determine.	No downgrade
Overall judgment			Very low quality
6. Dose–response	Significant trend	Not possible to assess	No reason for upgrade
7. Magnitude of effect	RR > 2	Not possible to assess	No reason for upgrade
8. Confounding adjusted	Effect in spite of confounding working towards the nil	Not possible to assess	No reason for upgrade
Overall Judgment			Very low quality

Assessment of the Risk of Bias in the Individual Studies

Table S16. Assessment of the **risk of bias** in studies in Table 3.

		Bias due to Selection of Participants	Information Bias due to Exposure Assessment	Outcome Bias I	Outcome Bias II	Bias due to Confounding	Count of Columns with Low Risk of bias	Total Risk of Bias
Brown [17]	2015	High	Low	Low	High	Low	3	High
Pedersen, Le Ray, Bendtsen & Kragh [18]	2013/14	High	Low	Low	High	High	2	High
Stansfeld, Haines, Berry & Burr [19]	2009	Low	High	High	High	Low	2	High
Baugham & Huddart [20]	1993	Unclear	High	High	High	High	0	High
Griffiths & Raw [21]	1989	Unclear	High	High	High	High	0	High
Brown [22]	1987	Low	Low	High	High	High	2	High
Griffiths & Raw [23]	1986	High	High	High	High	High	0	High
Brown, Hall & Kyle-Little [24]	1985	Low	High	High	High	Low	2	High
Langdon & Griffiths [25]	1982	Unclear	High	High	High	High	0	High
Kastka [26]	1981	Unclear	Unclear	High	High	High	0	High

Table S17. Assessment of the **risk of bias** in studies in Table 4.

		Bias due to Selection of Participants	Information Bias due to Exposure Assessment	Outcome Bias I	Outcome Bias II	Bias due to Confounding	Count of Columns with Low Risk of Bias	Total Risk of Bias
Amundsen, Klaeboe & Aasvang [27]	2011	High	Low	Low	High	Low	3	High
Bendtsen, Michelsen & Christensen [29]	2011	High	High	High	High	High	0	High
Gidlöf-Gunnarsson, Öhrström & Kihlman [30]	2010	High	High	Low	High	High	1	High
Nilsson & Berglund [32]	2006	Low	Low	High	High	High	2	High
Kastka, Buchta, Ritterstaedt, Paulsen & Mau [31]	1995	Low	Low	High	High	High	2	High
Vincent & Champelovier [33]	1993	High	High	High	High	High	0	High

Table S18. Assessment of the **risk of bias** in studies in Table 5.

		Bias due to Selection of Participants	Information Bias due to Exposure Assessment	Outcome Bias I	Outcome Bias II	Bias due to Confounding	Count of Columns with Low Risk of bias	Total Risk of Bias
Gidlöf-Gunnarsson, Svensson, & Öhrström [8]	2013	High	High	Low	High	High	1	High
Öhrström [7]	2004	Low	High	Low	High	High	2	High

Table S19. Assessment of the **risk of bias** in studies in Table 6.

		Bias due to Selection of Participants	Information Bias due to Exposure Assessment	Outcome Bias I	Outcome Bias II	Bias due to Confounding	Count of Columns with Low Risk of Bias	Total Risk of Bias
de Kluizenaar et al [35]	2013	High	Low	Low	High	Low	3	High
Babisch et al [36]	2012	High	Low	Low	High	Low	3	High
van Renteghem & Botteldooren [37]	2012	Unclear	Low	Low	High	Low	3	High
de Kluizenaar et al [38]	2011	Low	Low	High	High	Low	3	High
Gidlöf-Gunnarsson, & Öhrström [30,39]	2010	High	Low	Low	High	Low	3	High
Gidlöf-Gunnarsson, & Öhrström [40]	2007	High	Low	Low	High	Low	3	High

Table S20. Assessment of the **risk of bias** in studies in Table 7.

		Bias due to Selection of Participants	Information Bias due to Exposure Assessment	Outcome Bias I	Outcome Bias II	Bias due to Confounding	Count of Columns with Low Risk of Bias	Total Risk of Bias
Stansfeld, Haines, Berry & Burr [19]	2009	Low	High	Low	High	Low	3	High

Table S21. **A**ssessment of the **risk of bias** in studies in Table 8.

		Bias due to Selection of Participants	Information Bias due to Exposure Assessment	Outcome Bias I	Outcome Bias II	Bias due to Confounding	Count of Columns with Low Risk of Bias	Total Risk of Bias
Amundsen, Klaeboe & Aasvang [28]	2013	High	Low	Low	High	Low	3	High
Bendtsen, Michelsen & Christensen [29]	2011	High	High	High	High	High	0	High

Table S22. **A**ssessment of the **risk of bias** in studies in Table 9.

		Bias due to Selection of Participants	Information Bias due to Exposure Assessment	Outcome Bias I	Outcome Bias II	Bias due to Confounding	Count of Columns with Low Risk of Bias	Total Risk of Bias
Öhrström [7]	2004	Low	High	Low	High	High	2	High
Öhrström & Skanberg [41]	2004	Low	High	Low	High	Low	3	High

Table S23. **A**ssessment of the risk of bias in study in Table 10.

		Bias due to Selection of Participants	Information Bias due to Exposure Assessment	Outcome Bias I	Outcome Bias II	Bias due to Confounding	Count of Columns with Low Risk of Bias	Total Risk of Bias
van Renteghem & Botteldooren [37]	2012	Unclear	Low	Low	High	Low	3	High

Table S24. **A**ssessment of the **risk of bias** in studies in Table 11.

		Bias due to Selection of Participants	Information Bias due to Exposure Assessment	Outcome Bias I	Outcome Bias II	Bias due to Confounding	Count of Columns with Low Risk of Bias	Total Risk of Bias
Babisch et al. [42-43]	2014	Unclear	Low	Low	Unclear	Low	3	Unclear
Babisch et al. [36]	2012	High	Low	Low	High	Low	3	High
Lercher et al [44]	2011	High	Low	High	High	Low	2	High
Bluhm et al [45]	2007	Low	Low	High	High	High	2	High

Table S25. **A**ssessment of the **risk of bias** in studies in Table 12.

		Bias due to Selection of Participants	Information Bias due to Exposure Assessment	Outcome Bias I	Outcome Bias II	Bias due to Confounding	Count of Columns with Low Risk of Bias	Total Risk of Bias
Asensio, Recuero, & Pavón [46]	2014	High	Unclear	Low	High	High	1	High

Table S26. Assessment of the **risk of bias** in studies in Table 13.

		Bias due to Selection of Participants	Information Bias due to Exposure Assessment	Outcome Bias I	Outcome Bias II	Bias due to Confounding	Count of Columns with Low Risk of Bias	Total Risk of Bias
Brink, Wirth, Schierz, Thomann & Bauer [47]	2008	Low	Low	Low	Nigh	Low	4	Low
Breugelmans et al. [48]	2007	High	Low	Low	High	Low	3	High
Fidell, Silvati, and Haboly [49]	2002	Low	Low	Low	High	High	3	High

Table S27. Assessment of the **risk of bias** in studies in Table 14.

		Bias due to Selection of Participants	Information Bias due to Exposure Assessment	Outcome Bias I	Outcome Bias II	Bias due to Confounding	Count of Columns with Low Risk of Bias	Total Risk of Bias
Breugelmans et al. [48]	2007	High	Low	Low	High	Low	3	High
Fidell, Silvati, and Haboly [49]	2002	Low	Low	Low	High	High	3	High

Table S28. Assessment of the **risk of bias** in studies in Table 15.

		Bias due to Selection of Participants	Information Bias due to Exposure Assessment	Outcome Bias I	Outcome Bias II	Bias due to Confounding	Count of Columns with Low Risk of Bias	Total Risk of Bias
Hygge, Evans & Bullinger [50]	2002	Low	Low	Low	High	Low	4	Low

Table S29. Assessment of the **risk of bias** in studies in Table 17.

		Bias due to Selection of Participants	Information Bias due to Exposure Assessment	Outcome Bias I	Outcome Bias II	Bias due to Confounding	Count of Columns with Low Risk of Bias	Total Risk of Bias
Lam & Au [52]	2008	Low	High	Low	High	High	2	High

Supplementary File 6

Hospital Noise and PLD/Music Venues/Other Sources Interventions

For interventions for some noise sources and for some settings, specific subpopulations were considered: viz. patients in hospitals, and (primarily) young people who use personal listening devices (PLDs) or attend music events. For the hospital subpopulation, sources were all sounds heard in a hospital ward. For the subpopulation of adolescents, the noise exposure was the sound delivered to the users' ears through the headphones of personal listening devices, or the exposure experienced when attending music events or similar. Table S30 shows the number of individual studies considered within each group; Table S31 lists the studies excluded on full-text reading.

Table S30. Number of Individual Studies within each Group (Noise Source x Outcome Measure x Intervention Type).

	**Number of Peer Reviewed Papers**	**# Non-Peer Reviewed Papers**	**Total Papers per Group**
**HOSPITAL SOURCES**			
**Outcome: Sleep Disturbance**			
B Path Intervention	1	-	1
**Outcome: Cardiovascular Effects**			
A Source Intervention	1	-	1
**PLD/MUSIC VENUE/OTHER SOURCES**			
**Outcome: Hearing Loss/Tinnitus**			
E Change in Behaviour Intervention	7	-	7

Table S31. Studies Excluded Based on Full-text Reading.

**HOSPITAL NOISE**	
Byers JF, Smyth KA. (1997) Effect of a music intervention on noise annoyance, heart rate, and blood pressure in cardiac surgery patients. Am J Crit Care. 6:183–91. [105]	This is a study in a cardio intensive care unit. It assessed the experimental effect of a music intervention on noise annoyance, heart rate and blood presure. Excluded because intervention overlay ICU sounds with additional music.
Kamdar, B., King, L, Collop, N., Sakamuri, S. Colantuoni, E., Neufeld, K., Bienvenu, O., Rowden, A., Touradji, P., Brower, R., & Dale M. (2013). The Effect of a Quality Improvement Intervention on Perceived Sleep Quality and Cognition in a Medical ICU. *Critical Care Medicine*, *43*(3), 800–809. [106]	Interventions included mult-faceted sleep-promoting interventions including some that would have reduced noise exposure. However these were not in terms of changes in levels. Perceived sleep quality was measured as were ratings of noise. ICU quality improvements were associated with significant

	reductions in perceived nighttime noise levels and a substantial decrease in delirium/coma. Paper excluded as little specific information on noise levels or effects of interventions on levels.
Monsén, M. G., & Edéll-Gustafsson, U. M. (2005). Noise and sleep disturbance factors before and after implementation of a behavioural modification programme. *Intensive and Critical Care Nursing, 21*(4), 208–219. [107]<br>Persson Waye, K. Elmenhorst, E-M., Croy, I & Pedersen,, E. (2013) Improvement of intensive care unit sound environment and analyses of consequences on sleep: an experimental study. Sleep Medicine, 14, 1334–1340. [108]	Inadequate objective recording of noise disturbance factors and noise levels – which were the outcome meausres of the noise intervention program.<br>Study reported amount of REM sleep in 'patients' sleeping subject to ICU noise level exposures. Randomised control trial with 70 subjects. Subjects exposed to ICU noise experienced less REM and shorter REM than control group. Study excluded because used health volunteers
Richardson, A., Thompson, A., Coghill, E., Chambers, I., & Turnock, C. (2009). Development and implementation of a noise reduction intervention programme: a pre- and postaudit of three hospital wards. *J Clin Nurs, 18*(23), 3316–3324. doi: http://dx.doi.org/10.1111/j.1365–2702.2009.02897.x [109]<br>Stanchina ML, Abu-Hijleh M, Chaudhry BK, Carlisle CC, Millman R (2005).The influence of white noise on sleep in subjects exposed to ICU noise.Sleep Med 6:423–8. [110]	Inadequate reporting of outcome measure – change in noise levels in wards as a consequence of a behavioura modification program for nursing staff<br><br>This study examined the effect of white noise on sleep fragmentation to people sleeping while exposed to ICU noise. White noise increased ICU noise levels from 58 to 61 dB. Excluded because 'patients' were healthy volunteers. Also excluded because of small sample size (n=4)
Topf M, Davis JE. Critical care unit noise and rapid eye movement (REM) sleep.(1993) Heart Lung, 22:252–8W [111]	Average ICU levels were 62dBA. Examined REM sleep of 'patients' before and after fitting them with ear plugs. Use of earplugs resulted in more rapid eye movement sleep. Study excluded becaused used healthy volunteers as 'patients'.
Wallace JC, Robins J, Alvord LS, Walker JM. (1999)The effect of earplugs on sleep measures during exposure to simulated intensive care unit noise. Am J Crit Care; 8:210–9. [112]	Studies effect of ICU noise, and peak-reduced ICU noise, on sleep. Sleep registered with polysomnography. IC noise led to more fragmented sleep, more arousals and more time awake. Effects of reduced maxima were minor. Excluded because study was on healthy subjects.

Evidence: Hospital Noise

Two individual studies on hospital noise interventions met the inclusion criteria. The sources were the sounds that were internally generated in hospital wards, particularly intensive care units or similar, such as equipment, alarms, doors, voices etc. The outcomes reported were those for patients, often intensive-care patients, in hospital wards, for sleep disturbance, cardiovascular and other effects.

Outcome: Sleep Disturbance

Summary: Evidence from path interventions, Table S32

This study reports the effect of the wearing, by ICU patients, of ear plugs to reduce noise exposure. The intervention was effective in reducing intensive care delirium and, after the first night of sleep in the ICU, improving the patients' perception of their own sleep.

This study ruled out confounding by matching patient groups on a range of demographic factors, lifestyle, illness, and environmental factors. Risk of bias was

assessed as low.

Table S32. PATH INTERVENTIONS (Type B).

Authors	Intervention & Study		N, Response Rate& Method	Exposure Levels	Change in levels	Outcome measure(s)	Did outcome change with change in exposure? Yes/No (significance tested?)	Measure of association/strength	Confounders adjusted for in analyses
	Nature	Design							
Van Rompaey et al (2012) [113]	ICU patients wearing earplugs at night to prevent intensive care delirium.	Randomized clinical trial	136//69-<br>Intervention group: 69<br>Control 67<br>Method: questionnaires<br>Response rates 62%.	ICU noise	Earplugs lower exposure by approx. 33 dB	Confusion/Delerium<br>Subjective sleep<br>NEECHAM scale (4 categories) and<br>5 dichotomous questions regarding sleep	**Yes**<br><br>See next column	Use of earplugs lowered incidence of confusion: Hazard Ratio of 0.47 (95% CI 0.27 to 0.82).<br>After first night in ICU, patients with earplugs reported better sleep perception.	Study design ruled out a range of demographic factors, lifestyle, illness, and environmental factors

Table S33. Assessment of the **risk of bias** in studies in Table S32.

		Bias due to selection of participants	Information bias due to exposure assessment	Outcome bias I	Outcome bias II	Bias due to confounding	Count of columns with low risk of bias	Total risk of bias
Van Rompaey et al [113]	2012	Low	n.a.	Low?	Low	Low	4	Low

Outcome: Cardiovascular Effects

Summary: Information from source interventions, Table S34

This study reports the effect of hospital noise reduction by use of noise absorbing tiles in an intensive Coronary Care Unit, focussing on the effect of the intervention on hospitalized myocardial patients. Heart Rate, Blood Pressure Pulse amplitude as well as perceptions were measured for two groups: one under good acoustic conditions (following the intervention) and one under bad acoustic conditions. The intervention resulted in a significant physiological effect (change in pulse amplitude) as well as several changes in perceptions of staff, attitude, etc. Remarkable was the utilization of objective physiological response to measure the effect of the intervention.

Table S34. SOURCE INTERVENTIONS (Type A).

Authors	Intervention & Study		N, & Method	Exposure Levels and changes from intervention	Outcome measure(s) Before and after outcomes	Did outcome change with change in exposure? Yes/No	Confounders adjusted for in analyses
	Nature	Design					
Hagerman et al (2005) [114]	Installation of sound absorbing tiles (good acoustics) in Coronary Care Unit.	Patients recruited to either 'good' or 'bad' acoustic situations.	31 P recruited to bad acoustics & 63 to good acoustics. Myocardial patients	L_{eq} (period not specified) Was 56/57 in work area, both before and after the intervention, but dropped 5 to 6 dB in patient rooms. Reverberation time dropped from 0.8/0.9s to 0.4s.	Heart Rate, Blood Pressure Pulse amplitude Patient perceptions re quality of care.	**YES**<br>T-tests and U tests (for non-parametric variables)<br>Pulse amplitude drops in 2 out of 3 patient *degree of disease* groups (p<.03 and p<.04)<br>Significant difference (improvements) between good and bad acoustic group (in both total and myocardial groups (p< .06 - .009) and in patient ratings, particularly their view of staff attitude.	Demographics, disease related (duration and number of times hospitalized etc.).

Table S35. Assessment of the **risk of bias** in studies in Table S34.

		Bias due to selection of participants	Information bias due to exposure assessment	Outcome bias I	Outcome bias II	Bias due to confounding	Count of columns with low risk of bias	Total risk of bias
Hagerman et al [114]	2005	Low	Low	Low	High	High	3	High

Evidence: PLD/Music Venues/Other Sources

Seven individual studies on Personal Listening Devices (PLDs), attendance at music venues, and participation in other recreational activities, where there was risk of hearing damage and/or tinnitus, met the inclusion criteria. For all studies, the interventions were aimed at children or adolescents, to change hearing damage risk behaviour, or knowledge of risk. The outcome assessed in all intervention studies was (change in) knowledge of, and behaviours towards, hearing damage risk. There were no objectively measured outcomes.

Note that all interventions examined in this section were of Type E (interventions directed at changes in knowledge or behaviour) and do not include a change in noise level exposure. These studies were not required to meet the general rule for all other individual studies of reporting a change in noise levels in order to be included.

Outcome: Knowledge/Attitude/Behaviour

Summary: Evidence from behavioural interventions, Table S36.

The studies all sought evidence on the effectiveness of some form of educational program/campaign on children, adolescents or college students on their

perceptions and knowledge of the risk of high levels of noise – generally but not exclusively from PLD sources or from attendance at music events – and on their actual or intended changes to hearing damage risk behaviours including avoidance, frequency or durations of exposures, including regeneration periods when in high noise, or playback levels. Most studies found a significant effect of change in knowledge or behaviour, but at least one author questions if the effects will persist.

Table S36. CHANGE IN BEHAVIOUR INTERVENTIONS (Type E).

Author (year)	Type of Intervention	Objective:	N & age	Design	Outcome Assessment	Confounders	Findings
Gilles & Van de Heyning (2014) [115]	Education campaign	Focussed on the harmful effects of recreational noise and use of hearing protection	547<br><br>14–18 yrs age	Health promotion study<br>BA design using questionnaire	Attitude towards noise and hearing protection measured by YANS (Youth Attitude towards Noise Scale) and BAHPHL (Beliefs about Hearing Protection and Hearing Loss Scale)	Not mentioned	Scores on YANS and BAHPHL decreased significantly (p<.001).<br>Significant increase 4–14%/ (p < .001) in use of hearing protection, in those students familiar with the campaign.
Taljaard, Leishman & Eikelboom (2013) [116]	Education about hearing and listening (Cheers for Ears Program)	Increased knowledge of noise impact of PLDs. Alter self-reported listening behaviour	318<br><br>Primary school 9–13 yrs	B and A study. Two post-intervention rounds. Surveys completed in class.	Knowledge about hearing damage by loud sound; PLD use	Not mentioned	Wilcoxon rank test,significant changes (<0.0001–.03) are reported in Ps knowledge about hearing and in listening behaviour of the participants as measured by pre-and post-measurement. Listening time to PLDs did not change, volume settings selected did reduce Knowledge and changes in behaviour stable at 3 mos.
Martin, Griest, Sobel, & Howarth (2013) [117]	Different forms of information in NIHL prevention programs	Knowledge, attitudes and intended hearing protection behaviour	1120<br>Fourth grade students	Randomized trial with a non-intervention control group. Three measurement points using questionnaire	Attitude toward noise and hearing protection and intended behaviours re exposure and protection.	Not mentioned	All interventions (museum exhibition, different classroom programs, internet-based) effective, but decrease over time.<br><br>Live presentation more effective than internet information.
Dell & Holmes (2012) [118]	Education/Hearing conservation programme.	Knowledge, attitudes towards exposures to high-intensity sounds	64<br>Adolescents<br>12–14 yrs	Pre and Post surveys associated with a hearing conservation program.	Attitude towards noise measured by Youth Attitudes towards Noise Scale	Gender, Race	Wilcoxon signed rank test showed reduced score on YANS.<br>Significant reduction (p <0.003) in pro-noise attitudes.
Kotowski, Smith, Johnstone & Pritt (2011) [119]	Deployment of brochure about hearing protection	Perceptions of hearing loss threat, knowledge of hearing protection efficacy, and intended hearing protection use	176<br><br>college students	Randomized two group post-test.<br><br>Questionnaire	Extended Parallel Process Model	Demographics; year in school; hearing loss.	Exposure to the brochure increased awareness. Also increased behavioural intentions to use over-the-ear headphones - not ear plugs.<br>In future study include moderator variable: desirability of performing the recommended response.

Weichbold & Zorowka (2003) [120]	Hearing education campaign	Discotheque and ear-plug behaviours	169<br><br>High school students	BA study<br>Before and 1 yr after education campaign.<br><br>Questionnaire	1) Frequency of discotheque attendance (2) earplugs at disco		The campaign had little effect on inducing hearing-protective behaviour in adolescents.
Weichbold & Zorowka (2007) [121]	Hearing education campaign,	Music listening behaviours	1757<br><br>High school students	BA study<br>Before and 1 yr after education campaign.<br>Questionnaire	(1) Frequency of discotheque attendance (2) duration at disco (3) earplugs at disco (4) freq. of regeneration breaks at disco (5) mean time per week PLD	age	No change in behaviours except: frequency of disco attendance increased post campaign; increase in number of regeneration breaks while at a disco.<br>Effectiveness of hearing education campaign is highly doubted by the authors

There is no assessment of the **risk of bias** in studies in Table S36.

Summary

It is noted that there were few studies for PLD/music venue and hospital settings. Table S37 provides an overview of the observed magnitude of change in health outcome as a result of these interventions. Nearly all entries in Table S37 show that most of interventions led to a change in the aggregate health outcome of those who experienced the intervention (asterisk shown in the YES column), irrespective of the source type and irrespective of the type of intervention.

Two of the available studies of PLD/music venue sources suggest that behavioural/educational interventions for young people with respect to hearing risk may not be sustainable.

Table S37. Summary of evidence from the individual intervention studies: Hospital sources and PLD/Music Venue sources.

	Number of Papers	Evidence that health outcome changed?		
		**YES**	**NO**	**n.a.**
**HOSPITAL SOURCES** (2)				
**Outcome: Sleep Disturbance** (1)				
B Path Intervention	1	*		
**Outcome: Cardiovascular Effects (**1)				
A Source Intervention	1	*		
**PLD/MUSIC VENUE SOURCES** (7)				
**Outcome: Change in Behaviour**				
E Education/Communication	7	*****	**	

* Statistical significance of finding reported in the original study; * Finding interpreted by original or current authors based on data/tables/plots in original study.

Table S38. GRADE Table for the quality of evidence for hospital interventions Type B (Path Interventions) and sleep disturbance.

Domains	Criterion	Assessment	Downgrading
Start Level	Model protocol for longitudinal study design for noise interventions is reported in Section 9.2.2 of systematic review of interventions = high	One controlled before-and-after study.	Moderate quality
1. Study Limitations	Risk of bias	Low risk of bias	No downgrade
2. Inconsistency	Conflicting results; high I^2	I^2 not possible to determine	Downgrade one level
3. Directness	Direct comparison; same PECCO	The studies assess the impact of interventions along the pathway between environmental noise and human health.	No reason for downgrading
4. Precision	Confidence interval contains 25% harm or benefit	Numerical results and CIs.	No downgrade
5. Publication Bias	Funnel plot indicates	Unclear, not possible to determine.	No downgrade
Overall judgment			Low quality
6. Dose–response	Significant trend	Not possible to assess	No reason for upgrade
7. Magnitude of effect	RR > 2	Not possible to assess	No reason for upgrade
8. Confounding adjusted	Effect in spite of confounding working towards the nil	Not possible to assess	No reason for upgrade
Overall Judgment			Low quality

Table S39. GRADE Table for the quality of evidence for hospital interventions Type A (Source Interventions) and cardiovascular effects.

Domains	Criterion	Assessment	Downgrading
Start Level	Model protocol for longitudinal study design for noise interventions is reported in Section 9.2.2 of systematic review of interventions = high	One study with patients recruited to different conditions	Moderate quality
1. Study Limitations	Risk of bias	High risk of bias	Downgrade one level
2. Inconsistency	Conflicting results; high I^2	I^2 not possible to determine	Downgrade one level
3. Directness	Direct comparison; same PECCO	The studies assess the impact of interventions along the pathway between environmental noise and human health.	No reason for downgrading
4. Precision	Confidence interval contains 25% harm or benefit	Numerical results and CIs.	No downgrade
5. Publication Bias	Funnel plot indicates	Unclear, not possible to determine.	No downgrade
Overall judgment			Very low quality
6. Dose–response	Significant trend	Not possible to assess	No reason for upgrade
7. Magnitude of effect	RR > 2	Not possible to assess	No reason for upgrade
8. Confounding adjusted	Effect in spite of confounding working towards the nil	Not possible to assess	No reason for upgrade
Overall Judgment			Very low quality

References

1. Brown, A.L.; van Kamp, I. A Conceptual Model of Environmental Noise Interventions and Human Health Effects. In Proceedings of the INTER-NOISE and NOISE-CON Congress and Conference, San Francisco, CA, USA, 9–12 August 2015.
2. National Research Council. *Estimating the Public Health Benefits of Proposed Air Pollution Regulations;* National Academies Press: Washington, DC, USA, 2002.
3. Group, H.A.W. *Assessing Health Impacts of Air Quality Regulations: Concepts and Methods for Accountability Research*; Health Effects Institute: Boston, MA, USA, 2003.
4. Van Erp, A.M.; Kelly, F.J.; Demerjian, K.L.; Pope, C.A.; Cohen, A.J. Progress in research to assess the effectiveness of air quality interventions towards improving public health. *Air Qual. Atmos. Heal*th **2012**, 5, 217–230.
5. Burns, J.; Boogaard, H.; Turley, R.L.; Pfadenhauer, L.M.; van Erp, A.M.; Rohwer, A.C.; Rehfuess, E. Interventions to reduce ambient particulate matter air pollution and their effect on health. Cochrane Library **2014**, doi:10.1002/14651858.CD010919.
6. Organization, W.H. *Guidelines for Community Noise*; WHO: Geneva, Switzerland, 1999.
7. Öhrström, E. Longitudinal surveys on effects of changes in road traffic noise—Annoyance, activity disturbances, and psycho-social well-being. *J. Acoust. Soc. Am*. **2004**, 115, 719–729.
8. Gidlöf-Gunnarsson, A.; Svensson, H.; Ohrstrom, E. Noise reduction by traffic diversion and a tunnel construction: Effects on health and well-being after opening of the Southern Link. In Proceedings of the INTER-NOISE and NOISE-CON Congress and Conference, Innsbruck, Austria, 15–18 Sepetember, 2013.
9. Koehler, J.; Ruijsbroek, A.; van Poll, R. Effectiveness of insulation measures and underlying factors. In Proceedings of the INTER-NOISE and NOISE-CON Congress and Conference, Honolulu, HI, USA, 3–6 December 2006.
10. Schultz, T.J. Synthesis of social surveys on noise annoyance. *J. Acoust. Soc. Am*. **1978**, 64, 377–405.
11. Federal Interagency Committee on Noise. Federal Agency Review of Selected Airport Noise Analysis Issues. Available online: https://fican1.files.wordpress.com/2015/10/reports_noise_analysis.pdf (accessed on 24 July 2017).
12. Miedema, H.M.; Vos, H. Exposure–response relationships for transportation noise. *J. Acoust. Soc. Am*. **1998**, 104, 3432–3445.
13. Miedema, H.; Oudshoorn, C. Annoyance from transportation noise: Relationships with exposure metrics DNL and DENL and their confidence intervals. *Environ. Health Perspect*. **2001**, 109, 409.
14. Miedema, H.; Oudshoorn, C. Position Paper on Dose Response Relationships between Transportation Noise and Annoyance. In *EU's Future Noise Policy WG2–Dose/Effect*; AIP Publishing: Melville, NY, USA, 2002; Volume 20.
15. Brown, A.L.; van Kamp, I. Response to a change in transport noise exposure: A review of evidence of a change effect. *J. Acoust. Soc. Am.* **2009**, 125, 3018–3029.
16. Horonjeff, R.D.; Robert, W.E. *Attitudinal Responses to Changes in Noise Exposure in Residential Communities*; NASA Langley Research Center: Hampton, VA, USA, 1997.
17. Brown, A.L. Longitudinal annoyance responses to a road traffic noise management strategy that reduced heavy vehicles at night. *J. Acoust. Soc. Am*. **2015**, 137, 165–176.
18. Pedersen, T.H.; Le Ray, G.; Bendtsen, H.; Kraugh, J. Community response to noise reducing road pavements. In Proceedings of the INTER-NOISE and NOISE-CON Congress and Conference, Innsbruck, Austria, 15–18 Sepetember, 2013.
19. Stansfeld, S.A.; Haines, M.M.; Berry, B.; Burr, M. Reduction of road traffic noise and mental health: An intervention study. *Noise Health* **2009**, 11, 169.
20. Baughan, C.; Huddart, L. Effects of traffic noise changes on residents' nuisance ratings. In Proceedings of the 6th International Congress on Noise as a Public Health Problem, Noise & Man, Nice, France, 5–9 July 1993; pp. 585–588.
21. Griffiths, I.; Raw, G. Adaptation to changes in traffic noise exposure. *J. Sound Vib*. **1989**, 132, 331–336.
22. Langdon, F.; Griffiths, I. Subjective effects of traffic noise exposure, II: Comparisons of noise indices, response scales, and the effects of changes in noise levels. *J. Sound Vib.* **1982**, 83, 171–180.
23. Brown, A.L. Responses to an increase in road traffic noise. *J. Sound Vib*. **1987**, 117, 69–79.
24. Griffiths, I.; Raw, G. Community and individual response to changes in traffic noise exposure. *J. Sound Vib.* **1986**, 111, 209–217.

25. Brown, A.L.; Hall, A.; Kyle-Little, J. Response to a reduction in traffic noise exposure. *J. Sound Vib.* **1985**, 98, 235-246.
26. Kastka, J. Zum Einfluss verkehrsberuhigender Maßnahmen auf Lärmbelastung und Lärmbelästigung. (The influence of traffic calming measures on noise load and noise annoyance). *ZfLärmbek* **1981**, 28, 25-30.
27. Amundsen, A.H.; Klæboe, R.; Aasvang, G.M. The Norwegian Façade Insulation Study: The efficacy of façade insulation in reducing noise annoyance due to road traffic. *J. Acoust. Soc. Am.* **2011**, 129, 1381–1389.
28. Amundsen, A.H.; Klæboe, R.; Aasvang, G.M. Long-term effects of noise reduction measures on noise annoyance and sleep disturbance: The Norwegian facade insulation study. *J. Acoust. Soc. Am.* **2013**, 133, 3921–3928.
29. Bendtsen, H.; Michelsen, L.; Christensen, E.C. Noise annoyance before and after enlarging Danish highway. Presented at the 6th Forum Acusticum, Aalborg, Denmark, 27 June–1 July 2011.
30. Gidlöf-Gunnarsson, A.; Öhrström, E.; Kihlman, T. A full-scale intervention example of the quiet-side concept in a residential area exposed to road traffic noise: Effects on the perceived sound environment and general annoyance. In Proceedings of the INTER-NOISE 2010 39th International Congress on Noise Control Engineering 2010, Lisbon, Portugal, 13–16 June 2010.
31. Kastka, J.; Buchta, E.; Ritterstaedt, U.; Paulsen, R.; Mau, U. The long term effect of noise protection barriers on the annoyance response of residents. *J. Sound Vib.* **1995**, 184, 823–852.
32. Nilsson, M.E.; Berglund, B. Noise annoyance and activity disturbance before and after the erection of a roadside noise barriera. *J. Acoust. Soc. Am.* **2006**, 119, 2178–2188.
33. Vincent, B.; Champelovier, P. Changes in the acoustic environment: Need for an extensive evaluation of annoyance. *Proc. Noise Man* **1993**, 93, 425–428.
34. Öhrström, E.; Skånberg, A. Adverse health effects in relation to noise mitigation–a longitudinal study in the city of Göteborg. In Proceedings of the The 29th International Congress and Exhibition on Noise Control Engineering, Nice, France, 27–30 August 2000; pp. 27–30.
35. De Kluizenaar, Y.; Janssen, S.A.; Vos, H.; Salomons, E.M.; Zhou, H.; van den Berg, F. Road traffic noise and annoyance: A quantification of the effect of quiet side exposure at dwellings. *Int. J. Environ. Res. Public Health* **2013**, 10, 2258–2270.
36. Babisch, W.; Swart, W.; Houthuijs, D.; Selander, J.; Bluhm, G.; Pershagen, G.; Dimakopoulou, K.; Haralabidis, A.S.; Katsouyanni, K.; Davou, E. Exposure modifiers of the relationships of transportation noise with high blood pressure and noise annoyance. *J. Acoust. Soc. Am.* **2012**, 132, 3788–3808.
37. van Renterghem, T.; Botteldooren, D. Focused study on the quiet side effect in dwellings highly exposed to road traffic noise. *Int. J. Environ. Res. Public Health* **2012**, 9, 4292–4310.
38. de Kluizenaar, Y.; Salomons, E.M.; Janssen, S.A.; van Lenthe, F.J.; Vos, H.; Zhou, H.; Miedema, H.M.; Mackenbach, J.P. Urban road traffic noise and annoyance: The effect of a quiet façade. *J. Acoust. Soc. Am.* **2011**, 130, 1936–1942.
39. Gidlöf-Gunnarsson, A.; Öhrström, E. Attractive "uiet" courtyards: A potential modifier of urban residents' responses to road traffic noise? *Int. J. Environ. Res. Public Health* **2010**, 7, 3359–3375.
40. Gidlöf-Gunnarsson, A.; Öhrström, E. Noise and well-being in urban residential environments: The potential role of perceived availability to nearby green areas. *Landsc. Urban Plan.* **2007**, 83, 115–126.
41. Öhrström, E.; Skånberg, A. Longitudinal surveys on effects of road traffic noise: Substudy on sleep assessed by wrist actigraphs and sleep logs. *J. Sound Vib.* **2004**, 272, 1097–1109.
42. Babisch, W.; Wölke, G.; Heinrich, J.; Straff, W. Road traffic noise and hypertension—Accounting for the location of rooms. *Environ. Res.* **2014**, 133, 380–387.
43. Babisch, W.; Wölke, G.; Heinrich, J.; Straff, W. Road Traffic, Location of Rooms and Hypertension. *J. Civ. Environ. Eng.* **2014**, 4, 1.
44. Lercher, P.; Botteldooren, D.; Widmann, U.; Uhrner, U.; Kammeringer, E. Cardiovascular effects of environmental noise: Research in Austria. *Noise Health* **2011**, 13, 234.
45. Bluhm, G.L.; Berglind, N.; Nordling, E.; Rosenlund, M. Road traffic noise and hypertension. *Occup. Environ. Med.* **2007**, 64, 122–126.
46. Asensio, C.; Recuero, M.; Pavón, I. Citizens' perception of the efficacy of airport noise insulation programmes in Spain. *Appl. Acoust.* **2014**, 84, 107–115.
47. Brink, M.; Wirth, K.E.; Schierz, C.; Thomann, G.; Bauer, G. Annoyance responses to stable and changing aircraft noise exposure. *J. Acoust. Soc. Am.* **2008**, 124, 2930–2941.
48. Breugelmans, O.; Houthuijs, D.; Van Kamp, I.; Stellato, R.; van Wiechen, C.; Doornbos, G. Longitudinal effects of a sudden change in aircraft noise exposure on annoyance and sleep disturbance around Amsterdam Airport. In Proceedings of the ICA, Madrid, Spain, 2–7 September 2007.

49. Fidell, S.; Silvati, L.; Haboly, E. Social survey of community response to a step change in aircraft noise exposure. J. *Acoust. Soc. Am.* **2002**, 111, 200–209.
50. Hygge, S.; Evans, G.W.; Bullinger, M. A prospective study of some effects of aircraft noise on cognitive performance in schoolchildren. *Psychol. Sci.* **2002**, 13, 469–474.
51. Möhler, U.; Hegner, A.; Schuemer, R.; Schuemer-Kohrs, A. Effects of railway-noise reduction on annoyance after rail-grinding. In Proceedings of the INTER-NOISE and NOISE-CON Congress and Conference, Honolulu, Budapest Hungary, 1997.
52. Lam, K.-C.; Au, W.-H. Human response to a step change in noise exposure following the opening of a new railway extension in Hong Kong. *Acta Acust. United Acust.* **2008**, 94, 553–562.
53. Schreckenberg, D.; Mohler, U.; Liepert, M.; Schuemer, R. The impact of railway grinding on noise levels and residents' noise responses - Part II: The role of information. In Proceedings of the INTER-NOISE and NOISE-CON Congress and Conference, Innsbruck Austria, 2013.
54. Brown, A.L.; van Kamp, I. Response to a change in transport noise exposure: Competing explanations of change effects. *J. Acoust. Soc. Am.* **2009**, 125, 905–914.
55. Tonne, C.; Beevers, S.; Armstrong, B.; Kelly, F.; Wilkinson, P. Air pollution and mortality benefits of the London Congestion Charge: Spatial and socioeconomic inequalities. *Occup. Environ. Med.* **2008**, 65, 620–627.
56. Cesaroni, G.; Boogaard, H.; Jonkers, S.; Porta, D.; Badaloni, C.; Cattani, G.; Forastiere, F.; Hoek, G. Health benefits of traffic-related air pollution reduction in different socioeconomic groups: The effect of low-emission zoning in Rome. *Occup. Environ. Med.* **2012**, 69, 133–139.
57. Laszlo, H. E.; McRobie, E. S.; Stansfeld, S. A.; Hansell, A. L. Annoyance and other reaction measures to changes in noise exposure - a review. *Science of the Total Environment* **2012**, 435-436 (October): 551-562.
58. van Kamp, I.; Brown, A.L. Response to change in noise exposure: an update. Proceedings of ACOUSTICS 2013, Conference of the Australian Acoustical Society, November,Victor Harbour, 2013.
59. Dravitzki, V. K.; Wood, C. W. B. Effects of road texture on traffic noise and annoyance at urban driving speeds. Paper presented at the 21st ARRB and 11th REAAA Conference, Transport Our Highway to a Sustainable Future, Cairns, QLD, 2003.
60. Walton, D.; Dravitzki, V. Community response to changes in noise from resurfaced roads. Paper presented at the 21st ARRB and 11th REAAA Conference, Transport Our Highway to a Sustainable Future, Cairns, QLD, 2003.
61. Eberhardt, J.L.; Akselson, K.R. The disturbance by road traffic noise of the sleep of young male adults as recorded in the home. *Journal of Sound and Vibration* **1987,** 114 (3): 417-434
62. Griefahn, B.; Marks, A.; Robens, S. Experiments on the time frame of temporally limited traffic curfews to prevent noise induced sleep disturbances. *Somnologie* **2008**, 12(2), 140-148.
63. Klæboe, R.; Kolbenstvedt, M.; Fyhri, A.; Solberg, S. The impact of an adverse neighbourhood soundscape on road traffic noise annoyance. *Acta Acustica United With Acustica* **2005,** 91, 1039–1050.
64. Gomez-Jacinto, L.; Moral-Toranzo, F. Urban traffic noise and self-reported health. *Psychological Reports* **1999**, 84, 1105-1108.
65. Harupa, A.; Richard, J. Expo project 'Quiet City' - Noise abatment planning in Hennigsdorf (Brandenburg). *Zeitschrift fur Larmbekampfung* **2000**, 47(6), 211-215.
66. Klæboe, R.; Kolbenstvedt, M.; Lercher, P.; Solberg, S. Changes in noise reactions – evidence for an area effect? In Proceedings of the INTER-NOISE and NOISE-CON Congress and Conference, 1998, Christchurch, New Zealand.
67. Mital, A.; Ramakrishnan, A. S. Effectiveness of noise barriers on an interstate highway: a subjective and objective evaluation. *J Hum Ergol* **1997**, 26(1), 31-38.
68. Öhrström E.; Björkman, M. Sleep disturbance before and after traffic noise attenuation in an apartment building. J. Acoust. Soc. Am. **1983**, 73(3): 877-9.
69. Tulen, J.H.M.; Kumar, A.; Jurriëns, A.A. Psychophysiological acoustics of indoor sound due to traffic noise during sleep. *J. Sound Vib,* **1986**, 110 (1): 129-142
70. Utley, W. A.; Buller, I. B.; Keighley, E. C.; Sargent, J. W. The Effectiveness and Acceptability of Measures for Insulating Dwellings against Traffic Noise. *J. Sound Vib*, **1986**, 109(1), 1-18.
71. Wilkinson, R.T. / Campbell, K.B. Effects of traffic noise on quality of sleep: Assessment by EEG, subjective report, or performance the next day. *J Acoust Soc Am*, **1984**, 75(2): 468-475
72. Cohen, S., et al. Aircraft noise and children: Longitudinal and cross-sectional evidence on adaptation to noise and the effectiveness of noise abatement. *Journal of Personality and Social Psychology*, **1981**, 40(2), 331-345. doi: 10.1037//0022-3514.40.2.331
73. Fidell, S.; Horonjeff, R.; Teffeteller, S.; Pearsons, K. Community sensitivity to changes in aircraft noise exposure. *Hutnik*, **1981**.

74. Fidell, S.; Pearsons, K.; Tabachnick, B. G.; Howe, R. Effects on sleep disturbance of changes in aircraft noise near three airports. *J Acoust Soc Am*, **2000**, 107(5 Pt 1), 2535-2547.
75. Klæboe, R.. Aircraft noise annoyance in recreational areas after changes in noise exposure: Comments on Krog and Engdahl (2004) (L). J Acoust Soc Am, *2005*, 118(3 I), 1265-1267.
76. Krog, N. H.; Engdahl, B. Annoyance with aircraft noise in local recreational areas, contingent on changes in exposure and other context variables. *J Acoust Soc Am*, **2004**, 116(1), 323-333.
77. Krog, N. H.; Engdahl, B. Annoyance with aircraft noise in local recreational areas and the recreationists' noise situation at home. *J Acoust Soc Am*, **2005**, 117(1), 221-231.
78. Seabi, J. An epidemiological prospective study of children's health and annoyance reactions to aircraft noise exposure in *South Africa. Int J Environ Res Public Health*, **2013**, 10(7), 2760-2777. doi: http://dx.doi.org/10.3390/ijerph10072760
79. Stansfeld, S.; Hygge, S.; Clark, C.; Alfred, T. Night time aircraft noise exposure and children's cognitive performance. *Noise Health*, **2010**, 12(49), 255-262. doi: http://dx.doi.org/10.4103/1463-1741.70504
80. Van Kamp, I.; Houthuijs, D.; Van Wiechen, C.; Breugelmans, O. Environmental noise and mental health: Evidence from the Schiphol monitoring program. In Proceedings of the INTER-NOISE and NOISE-CON Congress and Conference, Istanbul, Turkey 2007.
81. Wirth, K.; Brink, M.; Schierz, C. Changes of community response to aircraft noise exposure over time. Paper presented at the EURONOISE 2006 - The 6th European Conference on Noise Control: Advanced Solutions for Noise Control, 2006.
82. Bronzaft A.L. The effect of a noise abatement program on reading ability. *J. Environ. Psychol.* **1981,** 1: 215–222.
83. Kawabata, T. Effects of Tohoku Shinkansen noise on living environment of school children--changes with the increase of the maximum train speed. *Nippon Koshu Eisei Zasshi,* **1991**, 38(1), 52-63.
84. Oka, S.; Tetsuya, H.; Yano, T.; Murakami, Y. Community response to a step change in railway noise and vibration exposures by the opening of a new Shinkansen Line. In Proceedings of the INTER-NOISE and NOISE-CON Congress and Conference, New York, New York, 2012.
85. Ohrstrom, E. Effects of exposure to railway noise - A comparison between areas with and without vibration. *J Sound Vib*, **1997**, 205 (4): 555-560.
86. Nykaza, E. T.; Pater, L. L.; Melton, R. H.; Luz, G. A. Minimizing sleep disturbance from blast noise producing training activities for residents living near a military installation. *J Acoust Soc Am*, **2009**, 125(1), 175-184. doi: http://dx.doi.org/10.1121/1.3026325
87. Steensberg, J. Community noise policy in Denmark. *J Public Health Policy*, **1999**, 20(1), 109-117.
88. Berge, T.; Storeheier, S. Å. Low noise pavements in a Nordic climate. Results from a four year project in Norway. In Proceedings of the INTER-NOISE and NOISE-CON Congress and Conference, Ottawa, Canada, 2009.
89. Brown, A. L.; Tomerini, D.; Carroll, J.; Scott, N. D. Non-responsiveness of conventional measures of road traffic noise to an urban truck restriction strategy. In Proceedings of the INTER-NOISE and NOISE-CON Congress and Conference, Ottawa, Canada, 2009.
90. Kim, S. K.; Park, W. J.; Lee, K. H. Noise reduction capacity of a composite pavement system. *KSCE Journal of Civil Engineering*, **2014**.
91. Khardi, S.; Abdallah, L.; Konovalova, O.; Houacine, M. Optimal approach minimizing aircraft noise and fuel consumption. *Acta Acustica united with Acustica*, **2010**, 96(1), 68-75.
92. Khardi, S.; Abdallah, L, Optimization approaches of aircraft flight path reducing noise: Comparison of modeling methods. *Applied Acoustics*, **2012**, 73 (4), 291-301.
93. Lakusic, S.; Ahac, M. Rail Traffic Noise and Vibration Mitigation Measures in Urban Areas. *Tehnicki Vjesnik-Technical Gazette*, **2012**, 19(2), 427-435.
94. Qing-fei, Z.; Si-jun, Z.; Lei, X.; Hai-ping, W.; Ming, Z.; Ming-sheng, L. Noise-reduction function and its affecting factors of urban plant communities in Shanghai. *Yingyong Shengtai Xuebao*, **2007**, 18(10), 2295-2300.
95. van Renterghem, T.; Attenborough, K.; Maennel, M.; Defrance, J.; Horoshenkov, K.; Kang, J.; . . . Yang, H. S. Measured light vehicle noise reduction by hedges. *Applied Acoustics*, **2014**, 78, 19-27. doi: 10.1016/j.apacoust.2013.10.011
96. Maris, E.; Stallen, P. J.; Vermunt, R.; Steensma, H. Noise within the social context: Annoyance reduction through fair procedures. *J Acoust Soc Am*, **2007**, 121(4), 2000-2010.
97. Lee, P. J.; Kim, Y. H.; Jeon, J. Y.; Song, K. D. Effects of apartment building facade and balcony design on the reduction of exterior noise. *Building and Environment*, **2007**, 42(10), 3517-3528.
98. Avsar, Y.; Gumus, B. D. The application of noise maps for traffic noise reduction. *Noise Control Engineering Journal*, **2011**, 59(6), 715-723.

99. Dintrans, A.; Préndez, M. A method of assessing measures to reduce road traffic noise: A case study in Santiago, Chile. *Applied Acoustics*, **2013**, 74(12), 1486-1491.
100. Giering, K.; Augustin, S. Effect-oriented index for railway noise. *Larmbekampfung*, **2011**, 6(4), 151-156.
101. Giering, K.; Augustin, S.; Strünke-Banz, S. Effect-related index for railway noise. In Proceedings of the INTER-NOISE and NOISE-CON Congress and Conference, Innsbruck, Austria, 2013.
102. Murphy, E.; King, E. A. Scenario analysis and noise action planning: Modelling the impact of mitigation measures on population exposure. *Applied Acoustics*, **2011**, 72(8), 487-494.
103. Roovers, M. S.; Van Blokland, G. J. Combined effects of source measures on road traffic noise annoyance in three major European cities. Paper presented at the Forum Acusticum Budapest 2005: 4th European Congress on Acustics.
104. Licitra, G.; Gagliardi, P.; Fredianelli, L.; Simonetti, D. Noise mitigation action plan of Pisa civil and military airport and its effects on people exposure. *Applied Acoustics*, **2014**, 84, 25-36.r
105. Byers, J.F.; Smyth, K.A. Effect of a music intervention on noise annoyance, heart rate, and blood pressure in cardiac surgery patients. *Am J Crit Care*. **1997**, 6:183-91.
106. Kamdar, B.; King, L.; Collop, N.; Sakamuri, S.; Colantuoni, E.; Neufeld, K.; Bienvenu, O.; Rowden, A.; Touradji, P.; Brower, R.; Dale M. The Effect of a Quality Improvement Intervention on Perceived Sleep Quality and Cognition in a Medical ICU. *Critical Care Medicine*, **2013**, 43(3), 800-809.
107. Monsén, M. G.; Edéll-Gustafsson, U. M. Noise and sleep disturbance factors before and after implementation of a behavioural modification programme. *Intensive and Critical Care Nursing*, **2005**, 21(4), 208-219.
108. Persson Waye, K.; Elmenhorst, E-M.; Croy, I.; Pedersen, E. Improvement of intensive care unit sound environment and analyses of consequences on sleep: an experimental study. *Sleep Medicine*, **2013**, 14, 1334-1340.
109. Richardson, A.; Thompson, A.; Coghill, E.; Chambers, I.; Turnock, C. Development and implementation of a noise reduction intervention programme: a pre- and postaudit of three hospital wards. *J Clin Nurs*, **2009**, 18(23), 3316-3324. doi: http://dx.doi.org/10.1111/j.1365-2702.2009.02897.x
110. Stanchina, M.L.; Abu-Hijleh, M.; Chaudhry, B.K.; Carlisle, C.C.; Millman, R. The influence of white noise on sleep in subjects exposed to ICU noise. *Sleep Med* **2005**, 6:423-8.
111. Topf, M.; Davis, J.E. Critical care unit noise and rapid eye movement (REM) sleep. *Heart Lung*, **1993**, 22:252-8W
112. Wallace, J.C.; Robins, J.; Alvord, L.S.; Walker, J.M. The effect of earplugs on sleep measures during exposure to simulated intensive care unit noise. *Am J Crit Care*; **1999**, 8:210-9.
113. Van Rompaey B.; Elseviers, M.; Van Drom, W.; Fromont, V.; Jorens, P. The effect of earplugs during the night on the onset of delirium and sleep perception: a randomized controlled trial in intensive care Patients, *Critical Care*, **2012**, 16:R73.
114. Hagerman, I. et al. Influence of intensive coronary care acoustics on the quality of care and physiological state of patients. *International Journal of Cardiology*, **2005**, 98, 267–270.
115. Gilles, A.; Van de Heyning, P. Effectiveness of a preventive campaign for noise-induced hearing damage in adolescents. *Int J Pediatr Otorhinolaryngol*, **2014**, 78(4), 604–609. doi: http://dx.doi.org/10.1016/j.ijporl.2014.01.009.
116. Taljaard, D. S.; Leishman, N. F.; Eikelboom, R. H. Personal listening devices and the prevention of noise induced hearing loss in children: the Cheers for Ears Pilot Program. *Noise Health*, **2013**, 15(65), 261–268. doi: http://dx.doi.org/10.4103/1463–1741.113523.
117. Martin, W. H.; Griest, S. E.; Sobel, J. L.; Howarth, L. C. Randomized trial of four noise-induced hearing loss and tinnitus prevention interventions for children. *Int J Audiol*, **2013,** 52 Suppl 1, S41–49. doi: http://dx.doi.org/10.3109/14992027.2012.743048.
118. Dell, S. M.; Holmes, A. E. The effect of a hearing conservation program on adolescents' attitudes towards noise. *Noise Health*, **2012**, 14(56), 39–44. doi: http://dx.doi.org/10.4103/1463–1741.93333.
119. Kotowski, M. R.; Smith, S. W.; Johnstone, P. M.; Pritt, E. Using the Extended Parallel Process Model to create and evaluate the effectiveness of brochures to reduce the risk for noise-induced hearing loss in college students. *Noise Health*, **2011**, 13(53), 261–271. doi: http://dx.doi.org/10.4103/1463–1741.82958.
120. Weichbold, V.; Zorowka, P. Effects of a hearing protection campaign on the discotheque attendance habits of high-school students. *Int J Audiol*, **2003**, 42(8), 489–493.
121. Weichbold, V.; Zorowka, P. Can a hearing education campaign for adolescents change their music listening behavior? *Int J Audiol*, **2007**, 46(3), 128–133.
